# Plant Virus‐Induced Inheritable Apoptosis Drives Reproductive Costs in Female Insect Vectors to Balance Viral Biparental Transmission

**DOI:** 10.1002/advs.202505681

**Published:** 2025-11-12

**Authors:** Haibo Wu, Wenqiang Wan, Qingquan Liang, Hengsong Yang, Chengcong Lu, Taiyun Wei, Qian Chen

**Affiliations:** ^1^ State Key Laboratory of Agricultural and Forestry Biosecurity, College of Plant Protection Fujian Agriculture and Forestry University Fuzhou 350002 China

**Keywords:** apoptosis, maternal transmission, PI3K/AKT/FoxO signaling, plant virus, reproductive costs

## Abstract

Insect‐borne pathogens often reduce the reproductive fitness of insect vectors. Rice gall dwarf virus (RGDV) is biparentally transmitted to the offspring of its leafhopper vector. However, maternal transmission of RGDV decreases female fecundity and disrupts egg development via an unknown mechanism. This study reveals that RGDV induces mitochondria‐dependent apoptosis in leafhopper ovaries, promoting viral infection but impairing ovary development. This apoptosis is transmitted to eggs during maternal RGDV transmission, promoting viral infection while harming eggs. RGDV in the ovary activates insulin‐like peptide‐driven PI3K signaling but reverses the downstream AKT/FoxO signaling axis. This reversal activates FoxO, which in turn transcribes pro‐apoptotic *Bcl‐2‐related ovarian killer*, triggering mitochondria‐dependent apoptosis. Moreover, RGDV capsid protein P2 is the key viral protein responsible for inducing apoptosis through the PI3K/AKT/FoxO signaling axis. Specifically, P2 initiates mitochondria‐dependent apoptosis by activating the PI3K signaling pathway upon recognition by insulin‐like peptide 2. Furthermore, P2 reverses the AKT/FoxO signaling axis via its interaction with PTEN. In contrast, two rice viruses, which are exclusively maternally transmitted, do not induce apoptosis in the ovary of insect vectors. These findings uncover how this biparentally transmitted plant virus induces inheritable apoptosis, thereby imposing female reproductive costs, and highlight viral manipulation of vector reproduction to optimize transmission.

## Graphical abstract

Rice gall dwarf virus (RGDV), transmitted biparentally by leafhopper vector to their offspring, exploits the capsid protein to induce inheritable ovarian apoptosis via insulin‐driven PI3K/AKT/FoxO signaling axis. This mechanism enhances infection but reduces female reproductive fitness, causing inefficient maternal transmission. RGDV spares male reproductive apoptosis and ensures efficient paternal transmission. This balanced strategy sustains viral persistence in China for 40+ years.

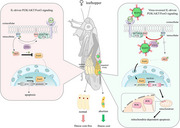

## 1 Introduction

Arthropods impact the health of plants, animals, and humans through the transmission of various pathogens. Generally, persistently transmitted pathogens establish mutualisms with arthropod vectors over their long‐term co‐evolution to ensure infection.^[^ ^1^, ^2^, ^3^ ^]^ However, some pathogens reduce the fitness of their arthropod hosts by impairing growth, development, and reproduction. Examples include rice stripe virus (RSV), southern rice black‐streaked dwarf virus, and tomato spotted wilt virus.^[^ ^4^, ^5^, ^6^, ^7^, ^8^, ^9^, ^10^ ^]^ In addition, some maternally transmitted microorganisms, including *Wolbachia*, *Spiroplasma*, *Rickettsia*, *Flavobacteria*, and γ‐proteobacteria, manipulate insect reproduction by altering offspring sex ratios, such as through male‐killing, which can sometimes benefit host populations by increasing the proportion of females.^[^ ^11^ ^]^ By contrast, cases in which mechanisms directly reduce the fitness of female vectors are rare. One such case is the pteromalus puparum negative‐strand RNA virus 1, which regulates offspring sex ratios by decreasing female offspring numbers.^[^ ^12^ ^]^ However, the mechanisms by which viruses impose fitness costs on female insect reproduction remain largely unclear.

Apoptosis, a form of programmed cell death, occurs under physiological homeostasis or in response to stress in all living organisms. During apoptosis, a cascade of caspase activation mediates apoptotic initiation, signal reception, and execution. Procaspase requires cleavage into ≈10–20 kDa in size to form active caspases.^[^ ^13^ ^]^ The inhibitor of apoptosis protein (IAP) can suppress excessive caspase‐dependent apoptosis, maintaining cell apoptosis to a normal extent.^[^ ^14^ ^]^ Some forms of apoptosis are mitochondria‐dependent and are initiated by abnormal expression of Bcl‐2 family proteins that localize to the mitochondrial membrane.^[^ ^15^ ^]^ Dysfunctional mitochondria promote the collapse of membrane potential and increase membrane permeability, leading to the release of apoptotic factors from mitochondria, such as cytochrome C, which initiates the apoptotic cascade in the cytoplasm. Apoptosis occurs in several organs, including the reproductive system of female animals.^[^ ^16^ ^]^ Apoptosis can be induced by viruses to inhibit viral replication and dissemination.^[^ ^17^, ^18^, ^19^ ^]^ For example, RSV decreases FAIM expression in its planthopper vector, thereby triggering an antiviral caspase‐dependent apoptosis in the midgut and salivary glands.^[^ ^20^ ^]^ However, whether viral infection can induce apoptosis in insect reproductive systems remains unknown.

The PI3K/AKT/FoxO signaling axis is a central pathway controlling growth and metabolism in all cells, and in vertebrates, it plays an important role in protecting cells from apoptosis.^[^ ^21^ ^]^ Class‐1 phosphatidylinositide 3‐kinases (PI3Ks), which act as anti‐apoptotic growth factors activated by numerous genes, phosphorylate phosphatidylinositol‐4,5‐bisphosphate (PIP2) to generate phosphatidylinositol‐3,4,5‐trisphosphate (PIP3). This reaction is reversed by phosphatase and tensin homolog (PTEN).^[^ ^22^ ^]^ PIP3 can recruit the downstream signaling protein serine‐threonine kinase AKT.^[^ ^23^ ^]^ Once activated, phosphorylated AKT prevents Forkhead box O (FoxO) transcription factors in the cytoplasm from their translocation to the nucleus and reduces their transcriptional activity, which regulates apoptosis and cell survival.^[^ ^23^ ^]^ In response to cell stimulation, decreased AKT activity allows phosphorylated FoxO to return to the nucleus, where it binds to target genes to induce transcription activation.^[^ ^24^ ^]^ This PI3K/AKT/FoxO signaling axis is regulated by a wide‐range of upstream signaling proteins, including insulin and insulin‐like peptides (ILPs).^[^ ^25^ ^]^ ILPs, which are among the most widely studied peptide hormones in insects, control germline stem cell proliferation and ovary development in female insects.^[^ ^26^ ^]^ ILPs activate the PI3K/AKT/FoxO signaling axis by binding to the insulin receptor (InR) on the cytomembrane. In insects, ILP‐driven PI3K/AKT/FoxO signaling axis functions as a potent regulator of cellular growth and metabolism, nutritional conditions, and female reproduction.^[^ ^27^ ^]^ However, it is unclear whether the ILP‐driven PI3K/AKT/FoxO axis controls apoptosis to regulate the reproductive development of insects.

Rice gall dwarf virus (RGDV) causes rice gall dwarf disease, which was first identified in Thailand and presently poses a highly destructive threat to rice production in southern China.^[^ ^28^ ^]^ This virus is a member of the genus *Phytoreovirus* in the family *Reoviridae*.^[^ ^29^ ^]^ RGDV is transmitted by the *Recilia dorsalis* (Hemiptera: Cicadellidae) leafhopper in a persistent‐propagative manner^[^ ^28^ ^]^ and can also be biparentally transmitted to leafhopper offspring.^[^ ^28^, ^30^, ^31^ ^]^ Notably, paternal transmission of RGDV is dominant and more effective under both field and laboratory conditions. RGDV hijacks surface proteins of sperms or cooperates with viral symbionts to hitchhike with sperms for paternal transmission without disturbing sperm functions.^[^ ^28^, ^31^ ^]^ The ribosome‐rescuer Pelo‐Hbs1 complex, which is involved in protein translational quality control, also functions as a sperm factor supporting paternal transmission.^[^ ^32^ ^]^ Immunofluorescence microscopy analysis of maternal transmission reveals a distinct spatial progression of RGDV infection within ovarian tissue. The virus initially invades the germarium, spreads through follicular cells toward the pedicel, and ultimately enters the developing oocyte, reaching the mature oocyte near the pedicel. Ultrastructural evidence from electron microscopy further corroborates this infection pattern, demonstrating active viral replication complexes within ovarian tissues.^[^ ^30^ ^]^ RGDV in ovarian follicular cells exploits virus‐containing tubules composed of the viral protein Pns11 to pass into oocytes, thus achieving infection of offspring.^[^ ^30^ ^]^ However, maternal transmission is slow and inefficient, and it also reduces female fitness, e.g., by shortening female longevity, decreasing egg number and hatching rates, and impairing egg development.^[^ ^28^, ^31^ ^]^ RGDV infection has also been reported to induce typical mitochondria‐dependent apoptosis in the alimentary canal and cultured cells of *R. dorsalis* to promote viral infection.^[^ ^33^ ^]^ It is unknown whether RGDV also induces ovary apoptosis, thus imposing a fitness cost on female reproduction.

In the present study, the mechanism by which RGDV imposes reproductive fitness costs on female leafhoppers was investigated. The virus was found to reverse ILP‐driven PI3K/AKT/FoxO signaling, thereby triggering downstream apoptosis and impacting insect reproduction. A key Bcl‐2 member controlling ovary virus‐induced apoptosis was identified, and this virus‐induced apoptosis was found to be transgenerationally inherited by eggs from females. Importantly, outer capsid protein P2 of RGDV was identified as the key apoptotic inducer in this process.

## 2 Results

### 2.1 RGDV Specifically Induces Mitochondria‐Dependent Apoptosis in Ovaries

**Figure 1:**
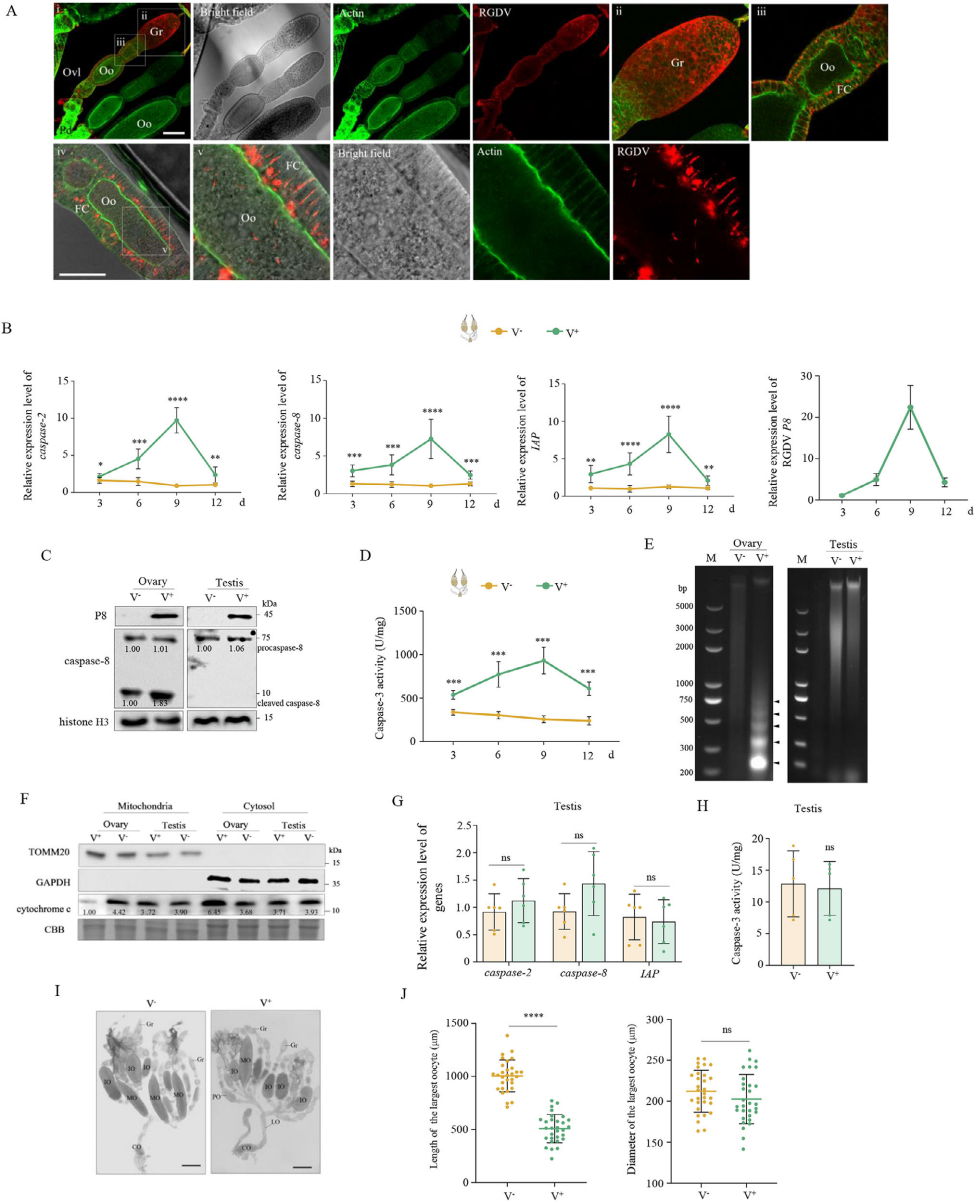
RGDV induces mitochondria‐dependent apoptosis in ovaries. A) Immunofluorescence microscopy showing RGDV infection in ovaries at 9 d after eclosion. Ovaries were stained with actin dye phalloidin‐FITC (green) and virus‐rhodamine (red). Panels ii, iii, and v show enlarged images of the boxed area in planels i and iv. Bars, 100 (i) and 50 (iv) µm. B) RGDV infection increased expression of *caspase‐2, caspase‐8*, and *IAP* in ovaries of females from 3 to 12 d after eclosion (*n _ovary_* = 30, at least four biological replicates). C) RGDV infection induced cleavage of caspase‐8 in ovaries at 9 d after eclosion (*n _ovary/testis_* = 20, three biological replicates). D) RGDV infection increased caspase‐3 activity of ovaries from 3 to 12 d after eclosion (*n _ovary_* = 30). E) RGDV infection induced chromosomal DNA fragmentation of ovaries at 9 d after eclosion (*n _ovary/testis_* = 40, three biological replicates). Lane M, DNA marker. F) RGDV infection promoted cytochrome C translocation from mitochondria to cytosol fractions of ovaries (*n _ovary/testis_* = 30, three biological replicates). Cytosol marker protein GAPDH and mitochondrial marker protein TOMM20 served as fraction controls. G, H) RGDV infection had a limited effect on the expression of *caspase‐2, caspase‐8*, and *IAP* (G) and caspase‐3 activity (H) in testes of males at 9 d after eclosion (*n _testis_* = 30). I, J) RGDV infection slowed the development of ovaries (*n _ovary_* = 1 in J). Bars, 300 µm. Fc, follicular cell. IO, immature oocyte. MO, mature oocytes. Oo, oocyte. Ovl, ovariole. Pd, pedicel. PO, primary oocyte. Gr, germarium. LO, lateral oviduct. CO, common oviduct. The relative intensities of bands for the proteins in C and F are shown below the corresponding bands. Bands of histone H3 indicate the loading of equal amounts of protein. Statistical analyses of B, D, G, H, and J were performed using Two‐tailed Student's *t*‐tests. Error bars show the SD. ^*^, *p* < 0.05; ^**^, *p* < 0.01; ^***^, *p* < 0.001; ^****^, *p* < 0.0001; ns, not significant.

We initially determined whether RGDV induces apoptosis in female ovaries. Immunofluorescence assays at 9 d after eclosion revealed that RGDV had infected germ cells throughout the germarium located at the anterior end of the ovariole, as well as the surrounding follicular cells of the oocyte (**Figure** **1**A‐i–iii). The virus also spread from the follicular cells into developing oocytes (Figure 1A‐vi–v). These findings are consistent with previous studies,^[^ ^30^ ^]^ confirming that the current experiments are well validated. RT‐qPCR assays revealed increased expression of *caspase‐2*, *caspase‐8*, and *IAP* genes in ovaries of RGDV‐infected (V^+^) females at 3, 6, 9, and 12 d after eclosion, relative to those of RGDV^−^free (V^−^) females (Figure 1B). Western blot assays showed that at 9 d after eclosion, RGDV infection triggered the maturation of caspase‐8, demonstrated as the increased accumulation of cleaved caspase‐8 (10 kDa) (Figure 1C). In addition, RGDV infection in ovaries improved caspase‐3 activity and caused the apoptotic DNA fragmentation evident from the formation of a typical DNA ladder, which was shown as non‐random fragments of 180–200 bp (Figure 1D,E). These results indicate that RGDV infection induces apoptosis in female ovaries.

Because apoptosis induced by RGDV infection in alimentary canals is mitochondria‐dependent,^[^ ^33^ ^]^ we examined whether RGDV‐induced apoptosis in ovaries was also mitochondria‐dependent. Mitochondria isolated from ovaries showed no detectable GAPDH, while the mitochondrial marker TOMM20 was absent from the cytosol (Figure 1F), indicating the purity of isolated mitochondria. The cytochrome C accumulation exhibited an increase in the cytosol derived from ovaries of V^+^ females compared with that of V^−^ females, along with a decrease in cytochrome C in the mitochondria (Figure 1F). This illustrates that RGDV infection in the ovaries induces cytochrome C release from mitochondria to the cytosol. Taken together, these results indicate that RGDV‐induced apoptosis in the ovary is also mitochondria‐dependent.

RGDV infection in testes at 9 d after eclosion had a limited effect on the gene expression or accumulation of caspase‐2, caspase‐8, and IAP, as well as on caspase‐3 activity (Figure 1C,G,H). Additionally, RGDV infection failed to cause DNA fragmentation and the redistribution of cytochrome C in testes (Figure 1E). Thus, RGDV infection does not induce apoptosis of the testes.

In contrast, the ovarian morphology and development of V^+^ females were analyzed. Generally, at 14 d after eclosion, most females were vitellogenic, and each ovary had one mature egg or one maturing oocyte (undergoing vitellogenesis) in each of its ovarioles.^[^ ^34^ ^]^ However, RGDV infection caused developmental retardation of ovaries, because most V^+^ ovarioles were previtellogenic (Figure 1I). The largest oocytes of V^+^ females were significantly smaller than those of V^−^ females (Figure 1J). These observations suggest that RGDV‐induced apoptosis likely impairs ovarian development.

### 2.2 Limited Apoptosis of Ovaries Benefits RGDV Infection

**Figure 2:**
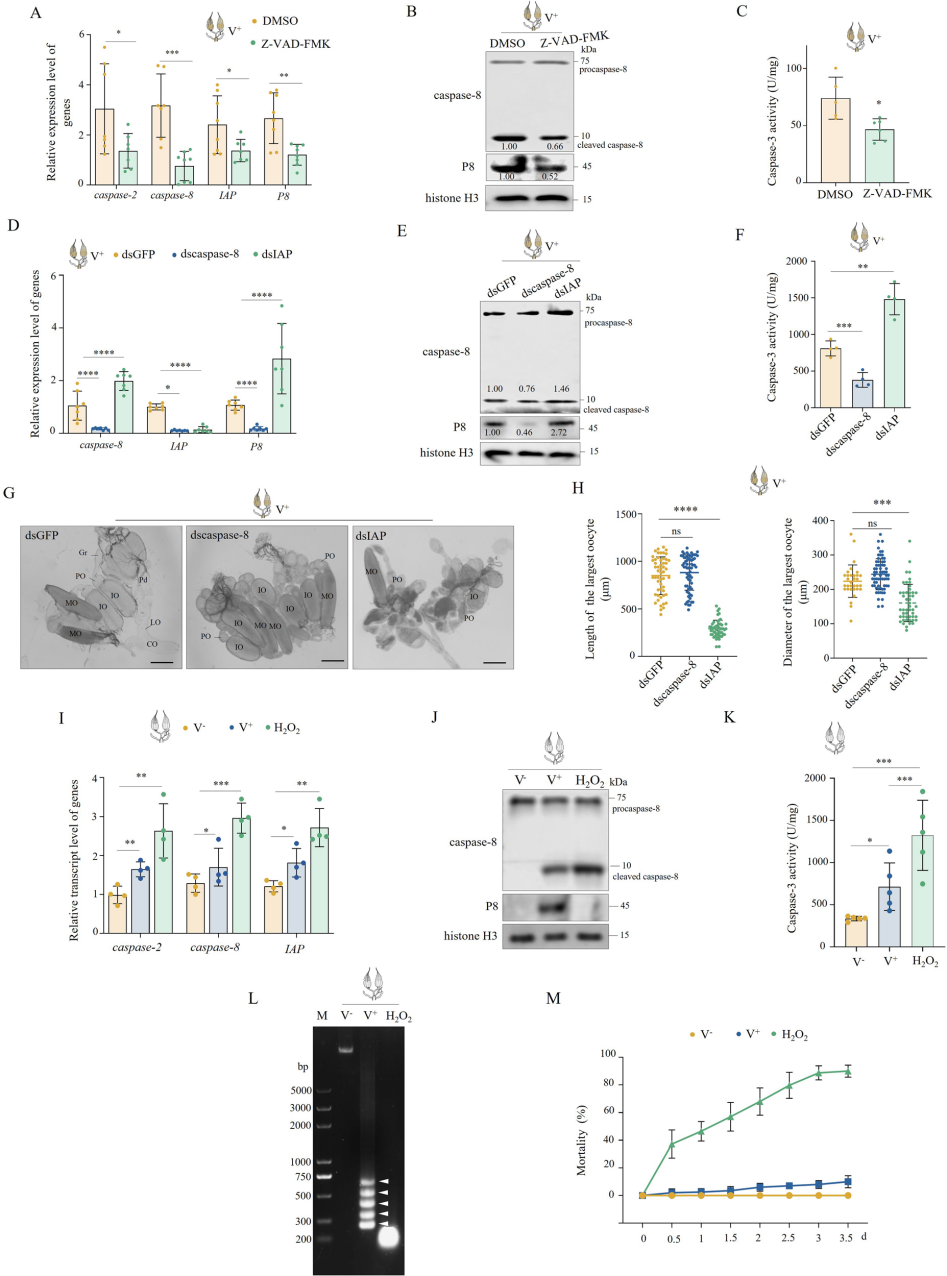
Limited apoptosis of the ovaries benefits RGDV infection. A) Z‐VAD‐FMK treatment reduced expression of *caspase‐2*, *caspase‐8*, *IAP*, and RGDV *P8* in ovaries derived from V^+^ females (*n _ovary_* = 30, at least seven biological replicates). B) Z‐VAD‐FMK treatment inhibited both activation of *caspase‐8* and RGDV infection in ovaries of V^+^ females (*n _ovary_* = 20, three biological replicates). C) Z‐VAD‐FMK treatment reduced caspase‐3 activity of ovaries of V^+^ females (*n _ovary_* = 30). D–F) Knocking down *caspase‐8* or *IAP* inhibited or promoted apoptosis and RGDV infection in ovaries (*n _ovary_* = 30 in D and F, *n _ovary_* = 20 in E, at least three biological replicates). G, H) Knocking down *caspase‐8* or *IAP* promoted or inhibited the development of ovaries derived from V^+^ females (*n _ovary_* = 1 in H). MO, mature oocytes; IO, immature oocyte; PO, primary oocyte; Gr, germarium; LO, lateral oviduct; CO, common oviduct. Bars, 300 µm. I–L) Apoptosis induced by RGDV was milder than that induced by 10% H_2_O_2_ in ovaries (*n _ovary_* = 30 in I and K, *n _ovary_* = 20 in J, *n _ovary_* = 40 in L, at least three biological replicates). Lane M, DNA marker. M) The mortality of females infected by RGDV was lower than that of females treated with 10% H_2_O_2_ (*n _female_* = 30, four biological replicates). The relative intensities of bands for the proteins in B, E, and J are shown above or below the corresponding bands. Bands of histone H3 indicate the loading of equal amounts of protein. Statistical analyses of A, C, D, F, H, I, K, and M were performed using Two‐tailed Student's *t*‐tests. Error bars show the SD. ^*^, *p* < 0.05; ^**^, *p* < 0.01; ^***^, *p* < 0.001.

To elucidate the biological significance of RGDV‐induced ovarian apoptosis, V+ females were treated with the broad‐spectrum caspase inhibitor Z‐VAD‐FMK, which exerts pan‐tissue inhibitory effects. At 3 d post Z‐VAD‐FMK treatment, the expression of *caspase‐2*, *caspase‐8*, *IAP*, and *P8* genes, accumulation of cleaved caspase‐8, and P8 proteins, as well as caspase‐3 activity, were all reduced (**Figure** **2**A–C). These results suggest that RGDV‐induced apoptosis in the ovary is caspase‐dependent. Knocking down *caspase‐8* also yielded similar results to Z‐VAD‐FMK treatment (Figure 2D–F). Additionally, knocking down *IAP*, which inhibits caspases,^[^ ^35^ ^]^ increased *caspase‐8* expression together with accumulation of procaspase‐8, cleaved caspase‐8, and P8 (Figure 2D–F). These results from Z‐VAD‐FMK treatment and knockdown of *caspase‐8* indicate that apoptosis in ovaries promotes RGDV infection.

The ovarian morphology and development of V^+^ females treated with dsRNAs were then analyzed. Knockdown of *caspase‐8* resulted in well‐stacked ovarioles with normally developed egg and oocytes, exhibiting limited effects on the size of the largest oocytes (Figure 2G,H). In contrast, knockdown of *IAP* caused most ovaries to be previtellogenic with small primary oocytes in each ovariole, reducing the size of the largest oocytes (Figure 2G,H). This suggests that RGDV‐induced apoptosis benefits viral infection but inhibits ovary development.

To determine the apoptotic level induced by RGDV infection, V^−^ females were treated with 10% H_2_O_2_, which induces apoptosis, at 9 d after eclosion. At 2 d post‐treatment, the expression levels of *caspase‐2*, *caspase‐8* and IAP, accumulation of procaspase‐8 and cleaved caspase‐8, and caspase‐3 activity of ovaries derived from V^+^ females were lower than those under H_2_O_2_ treatment (Figure 2I–K). DNA fragmentation of ovaries and mortality caused by RGDV were also respectively less severe and lower than those observed under 10% H_2_O_2_ treatment (Figure 2L–M). These results suggest that RGDV induces limited apoptosis, which restricts leafhopper mortality and consequently facilitates the maintenance of persistent infection in the leafhopper population.

### 2.3 Transgenerational Apoptosis Inherited from Female Promotes RGDV Infection in Offspring

**Figure 3:**
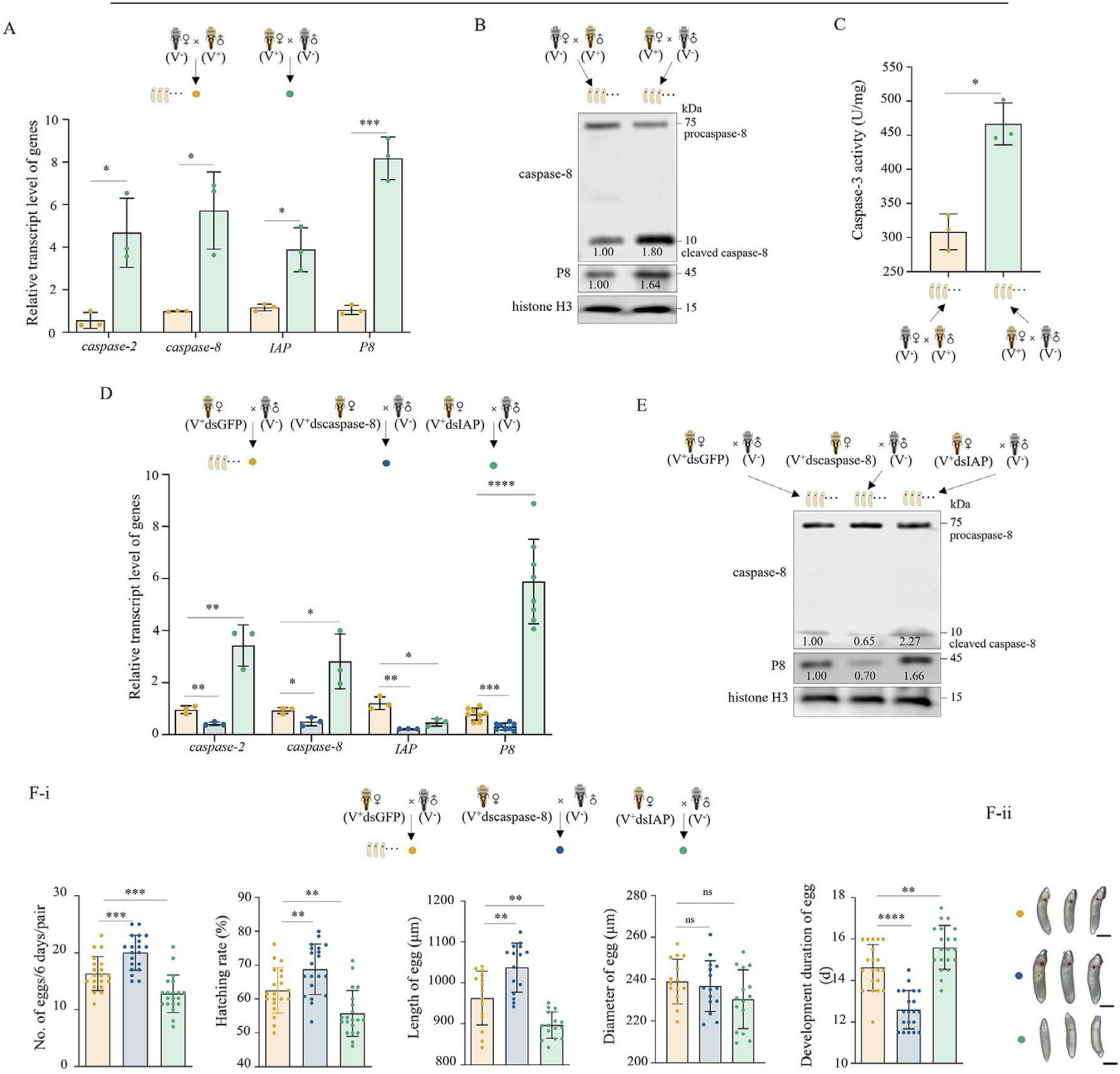
Transgenerational apoptosis from the female promotes RGDV infection in offspring. A–C) Maternally transmitted RGDV induced expression of *caspase‐2*, *caspase‐8*, and *IAP* genes, activation of caspase‐8, and activity of caspase‐3 in eggs (*n _egg_* = 10 in A; *n _egg_* = 50 produced by five mating pairs in B and C, at least three biological replicates). D, E) Knocking down *caspase‐2* or *IAP* in V^+^ females inhibited or promoted apoptosis in eggs (*n _egg_* = 10 in D; *n _egg_* = 50 produced by five mating pairs in E). F) Knocking down *caspase‐2* or *IAP* in V^+^ females increased or reduced the number, hatching rate, size, and development of progeny eggs (*n*_pair_ = 1, at least 15 biological replicates). The statistical results are displayed in F‐i. Representative egg phenotypes are displayed in F‐ii. Bars, 300 µm. Statistical analyses of A, C, D and F‐i were performed using Two‐tailed Student's *t*‐tests. Error bars show the SD. ^*^, *p* < 0.05; ^**^, *p* < 0.01; ^***^, *p* < 0.001; ^****^, *p* < 0.0001; ns, not significant.

To determine whether apoptosis is transgenerationally inherited from female parents, eggs from two mating pairings, (1) V^+^ virgin female × V^−^ male and (2) V^−^ virgin female × V^+^ male crosses, were analyzed. At 4 d post‐oviposition, eggs from V^+^ virgin female × V^−^ male crosses showed higher expression of *caspase‐2*, *caspase‐8*, and *IAP* genes, increased accumulation of cleaved caspase‐8 and P8 proteins, and elevated caspase‐3 activity compared to eggs from V^−^ virgin female × V^+^ male crosses (**Figure** **3**A–C). Knocking down *caspase‐8* in V^+^ virgin females, followed by mating with V^−^ males, resulted in reduced expression of *caspase‐2*, *caspase‐8*, and *IAP* genes and decreased accumulation of cleaved caspase‐8 and P8 in V^+^ eggs, compared to V^+^ eggs produced by dsGFP‐treated V^+^ virgin female × V^−^ male crosses (Figure 3D,E). In contrast, knocking down *IAP* in V^+^ virgin females caused increased expression of *caspase‐2*, *caspase‐8*, and *IAP* genes and higher accumulation of cleaved caspase‐8 and P8 proteins in V^+^ eggs (Figure 3D,E). These results indicate that RGDV‐induced apoptosis can be transgenerationally inherited, thus enhancing RGDV infection in eggs.

Biological assays showed that knocking down *caspase‐8* in V^+^ females increased the number, hatching rate, and size of eggs, and promoted egg development to the red‐eye stage after mating with V^−^ males (Figure 3F). However, knocking down *IAP* in V^+^ females yielded the opposite results (Figure 3F). In the context of previous results showing that transovarial transmission of RGDV significantly reduces female fecundity,^[^ ^28^ ^]^ these data indicate that transgenerationally inherited apoptosis passed on by females promotes viral infection, but also leads to offspring maldevelopment.

### 2.4 RGDV Triggers but Reverses the ILP‐Driven PI3K/AKT/FoxO Signaling Axis to Induce Ovarian Apoptosis

**Figure 4:**
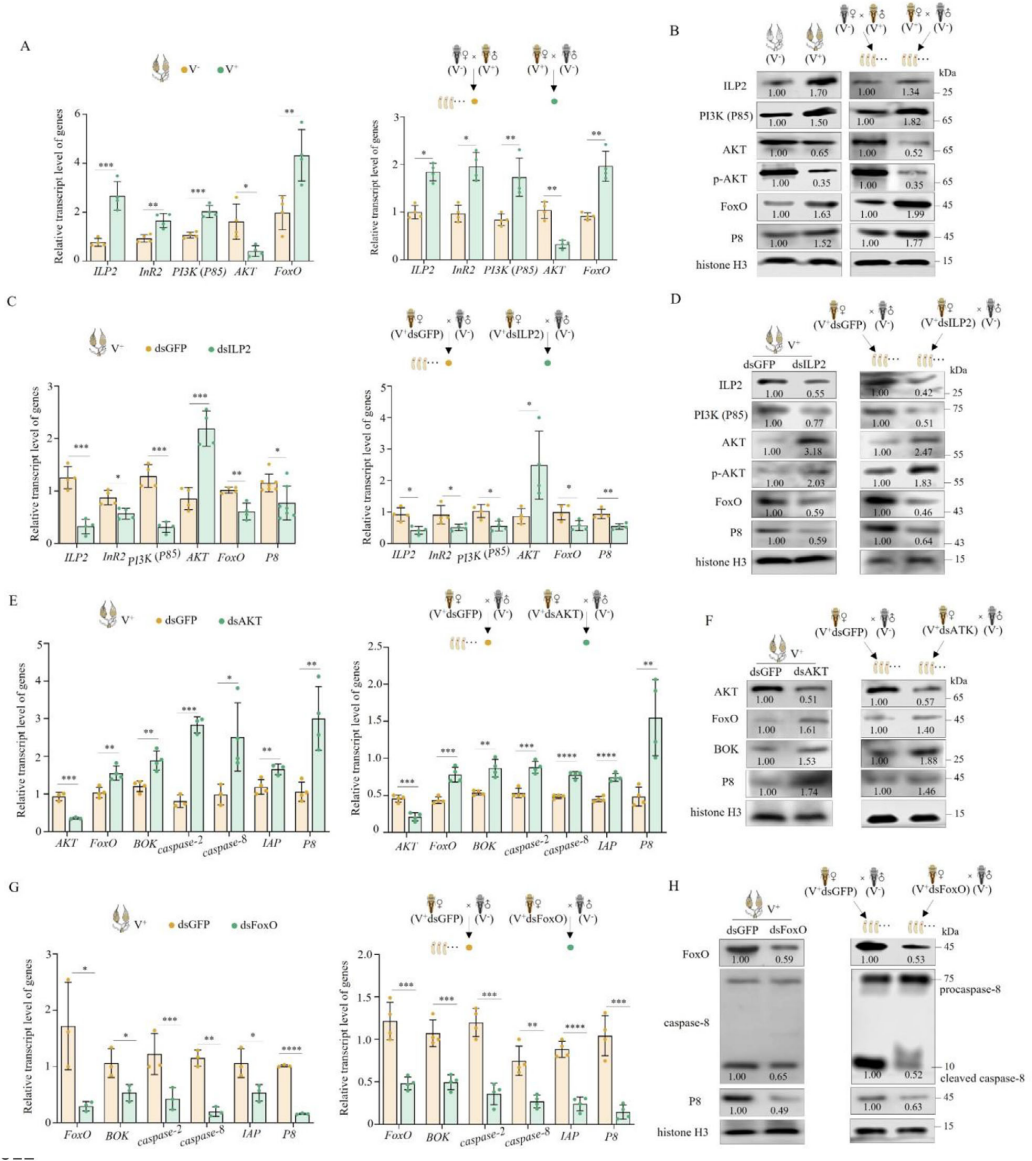
RGDV infection activates ILP‐driven PI3K‐AKT signaling to trigger apoptosis. A) RGDV infection of females increased expression of *ILP2*, *InR2*, *P85* (PI3K), and *FoxO* genes but reduced expression of *AKT* in ovaries and eggs (*n*_ovary_ = 30, *n*_egg_ = all eggs produced by four pairs of each mating combination, four biological replicates of each). B) RGDV infection of females increased accumulation of ILP2, P85, and FoxO, but reduced accumulation of AKT and p‐AKT in ovaries and eggs (*n*_ovary_ = 20, *n*_egg_ = all eggs produced by 12 pairs of each mating combination, three biological replicates of each). C–F) Knocking down *ILP2* and *AKT* in females promoted FoxO signaling and RGDV infection in ovaries and eggs produced by these females after mating with V^−^ males, as determined by RT‐qPCR (C, E) and western blot (D, F) assays. G, H) Knocking down *FoxO* in females inhibited apoptosis and RGDV infection in ovaries and eggs (*n*_ovary_ = 30, *n*_egg_ = all eggs produced by four pairs of each mating combination, four biological replicates in C, E, and G; *n*_ovary_ = 20, *n*_egg_ = all eggs produced by 12 pairs of each mating combination, three biological replicates in D, F, and H). The relative intensities of bands for the proteins in B, D, F, and H are shown below the corresponding bands. Bands of histone H3 indicate the loading of equal amounts of protein. Statistical analyses of A, C, E, and G were performed using Two‐tailed Student's *t*‐tests. Error bars show the SD. ^*^, *p* < 0.05; ^**^, *p* < 0.01; ^***^, *p* < 0.001; ^****^, *p* < 0.0001.

To address the mechanism of RGDV specifically inducing ovarian apoptosis, we analyzed the expression of genes related to insect reproduction, including *ecdysone receptor* (*EcR*) and *ecdysone‐inducible protein E75* (*E75*), which are related to ecdysone, *methoprene‐tolerant* (*Met*), and *Kruppel homolog1* (*Kr‐h1*), which are related to juvenile hormone, as well as ILPs. RGDV infection had limited effects on the expression of juvenile hormone‐related genes as well as *insulin‐ like peptide 1* (*ILP1*) and *insulin‐like peptide 4* (*ILP4*) expression, but it reduced the expression of ecdysone‐related genes in ovaries (Figure S1, Supporting Information). In contrast, RGDV infection increased the gene expression or accumulation of insulin‐like peptide 2 (ILP2) and its receptor InR (**Figure** **4**A,B). A similar pattern was observed in the V^+^ eggs produced by V^+^ virgin female × V^−^ male crosses, compared to the V^+^ eggs produced by V^−^ virgin female × V^+^ male crosses (Figure 4A,B). These results suggest that RGDV probably regulates female reproduction and egg development via the ILP pathway.

Insulin signaling is known to activate the downstream PI3K/AKT/FoxO signaling axis to reduce apoptosis and enhance cell proliferation.^[^ ^25^ ^]^ Although this previous conclusion contradicts what was found in this study, the key genes related to PI3K/AKT/FoxO signaling were analyzed. RGDV infection significantly increased the gene expression of *P85*, which is the heterodimeric PI3K regulatory subunit,^[^ ^36^ ^]^ and *FoxO*, but decreased *AKT* expression in ovaries of V^+^ females and the V^+^ eggs produced by V^+^ virgin female × V^−^ male crosses compared to V^−^ females and the eggs produced by V^−^ virgin female × V^+^ male crosses (Figure 4A). RGDV analysis showed increased accumulation of P85 and FoxO, but decreased the accumulation of AKT and phosphorylated AKT (p‐AKT) in the ovaries of V^+^ females and the V^+^ eggs produced by V^+^ virgin female × V^−^ male crosses (Figure 4B). These findings suggest the RGDV infection activates PI3K signaling in ovaries and offspring, while blocking the downstream ATK signaling axis.

Then, we examined the roles these key proteins play in RGDV‐induced apoptosis in the ovaries of V^+^ females or in V^+^ eggs produced by V^+^ virgin female × V^−^ male crosses. Knocking down *ILP2*, *InR2*, or *PI3K* (*P85*) of V^+^ females during the initial infection of RGDV in ovaries reduced the gene expression levels and the accumulation of ILP2, PI3K (P85), FoxO, or RGDV P8 proteins, but increased the expression or accumulation of AKT or p‐AKT in ovaries and V^+^ eggs produced by these females after mating with V^−^ males (Figure 4C–D; Figure S2, Supporting Information). In contrast, knocking down *AKT* decreased the accumulation of AKT but increased the gene expression or accumulation of FoxO, caspase‐2, caspase‐8, IAP, or P8 in the ovaries of V^+^ females and V^+^ eggs produced by these females after mating with V^−^ males (Figure 4E–F). Knocking down *FoxO* reduced the gene expression and accumulation of caspase‐2, caspase‐8, and RGDV P8, as well as the accumulation of cleaved caspase‐8 in ovaries of V^+^ females and V^+^ eggs produced by these females after mating with V^−^ males (Figure 4G–H). These results indicate that RGDV infection likely triggers ILP‐driven PI3K signaling while subsequently reversing the AKT/FoxO signaling axis, ultimately leading to apoptosis in ovarian tissues and eggs.

### 2.5 RGDV Infection Induces FoxO to Drive BOK Transcription, thus Inducing Apoptosis in Ovaries

**Figure 5:**
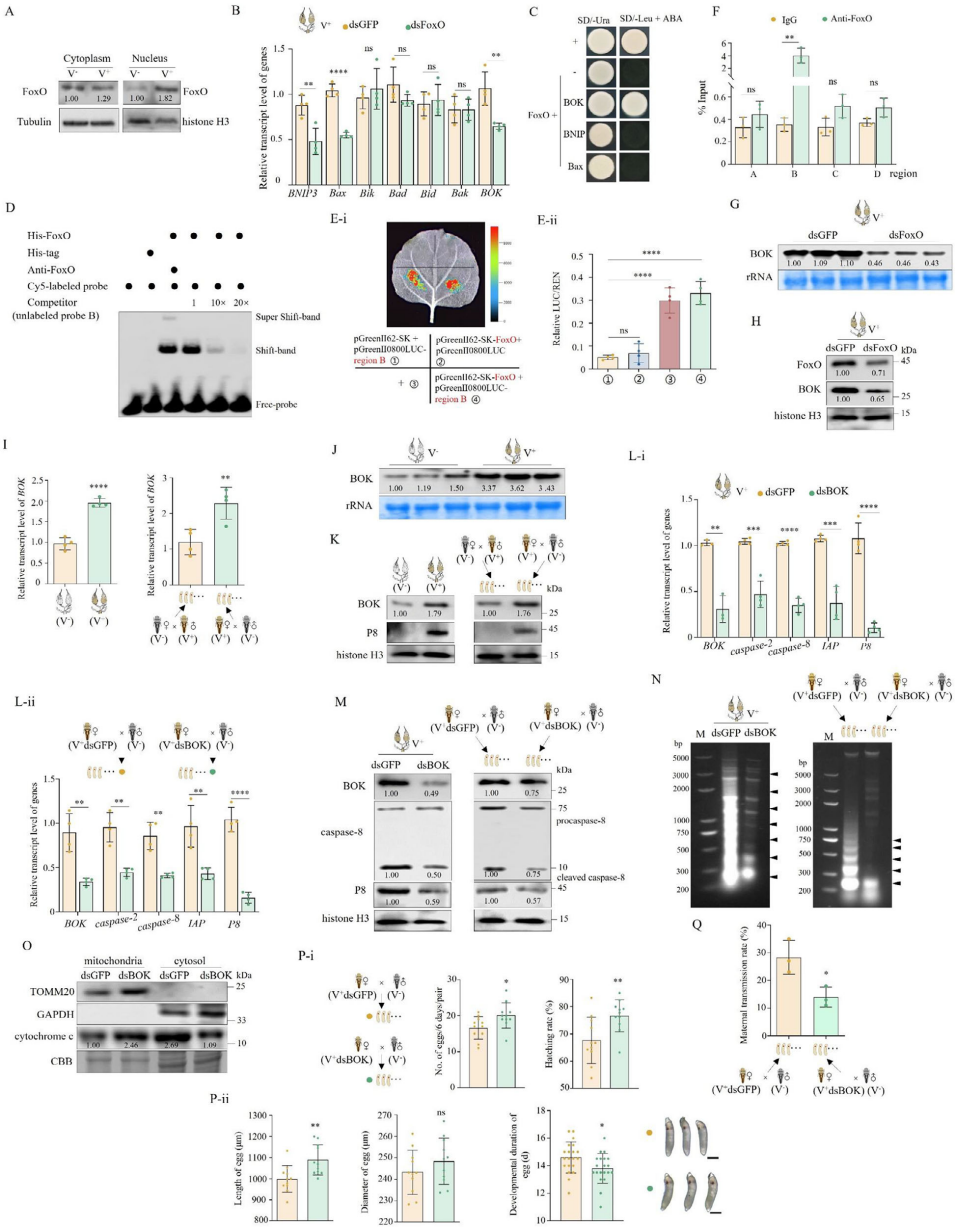
RGDV infection promotes FoxO‐mediated transcription of *BOK*, inducing ovarian apoptosis. A) RGDV infection induced FoxO translocation to nuclei in ovaries (*n*_ovary_ = 20, three biological replicates). B) Knocking down *FoxO* reduced the expression of *BNIP3*, *Bax*, and *BOK* genes in ovaries of V^+^ females (*n*_ovary_ = 30, *n*_egg_ = all eggs produced by four pairs of each mating combination, four biological replicates). C) Yeast one‐hybrid assay showing the affinity of FoxO and the 5′ non‐coding region (‐2000/‐1 bp) of *BOK*. +, positive control, i.e., pAbAi‐p53/pGADT7‐p53; ‐, negative control, i.e., pAbAi/pGADT7. D) EMSAs showing the binding of FoxO to promoter region B (‐858/‐849 bp) of *BOK*. The biotin‐labeled probe specific for region B served as the blank control, while the incubation of His‐tag with the probe served as the negative control. E) Dual‐luciferase reporter assays showing the FoxO‐activating region B of *BOK*. +, positive control, i.e., pGreenII62‐SK +pGreenII0800LUC‐35S. The luciferase (LUC) activity was measured as the ratio of LUC/REN (e‐II) (*n* = 1, three biological replicates). F) The qPCR assays of ChIP in vivo for the binding of FoxO to the *BOK* promoter. Fold enrichment was calculated after normalizing to 1% input (*n* = 1, three biological replicates). G) Northern blot assays showing knocking down *FoxO* of V^+^ females reduced transcription of *BOK* in ovaries (*n*_ovary_ = 20, three biological replicates). The rRNA samples stained with methylene blue served as the control to confirm loading of equal amounts of RNA in each lane. H) Knocking down *FoxO* in V^+^ females reduced expression of BOK in ovaries (*n*_ovary_ = 20, with three biological replicates). I) RGDV infection of females increased expression of *BOK* in ovaries and eggs (*n*_ovary_ = 30, *n*_egg_ = all eggs produced by four pairs of each mating combination, four biological replicates). J) Northern blot assays showing RGDV infection of females increased transcription of *BOK* in ovaries (*n*_ovary_ = 20, three biological replicates). K) RGDV infection increased accumulation of BOK in ovaries and eggs (*n*_ovary_ = 20, *n*_egg_ = all eggs produced by 12 pairs of each mating combination, three biological replicates of each). L, M) Knocking down *BOK* in V^+^ females reduced the gene expression and accumulation of BOK, caspase‐2, caspase‐8, IAP, and P8 in ovaries and eggs (*n*_ovary_ = 30, *n*_egg_ = all eggs produced by four pairs of each mating combination, four biological replicates in L; *n*_ovary_ = 20, *n*_egg_ = all eggs produced by 12 pairs of each mating combination, three biological replicates in m). N) Knocking down *BOK* in V^+^ females reduced chromosomal DNA fragmentation of ovaries and eggs (*n*_ovary_ = 40, *n*_egg_ = all eggs produced by five pairs of each mating combination, three biological replicates). Lane M, DNA marker. O) Knocking down *BOK* in V^+^ females inhibited cytochrome C translocation from mitochondria to cytosol fractions of ovaries. Cytosol GAPDH and mitochondrial TOMM20 served as fraction controls (*n*_ovary_ = 30, three biological replicates). P) Knocking down *BOK* in V^+^ females improved progeny egg number, hatching rate, and size, and extended the duration of egg development (*n*_pair_ = 1, 11 biological replicates). Q) Knocking down *BOK* in V^+^ females decreased the maternal transmission rate (*n*_pair_ = 1, four biological replicates). The relative intensities of bands for the proteins or genes in A, G, H, J, K, M, and O are shown below the corresponding bands. Cytoplasmic tubulin and nuclear histone H3 proteins served as fraction controls. Statistical analyses of B, E‐ii, F, I, L, P, and Q were performed using Two‐tailed Student's *t*‐tests. Error bars show the SD. ^*^, *p* < 0.05; ^**^, *p* < 0.01; ^***^, *p* < 0.001; ^****^, *p* < 0.0001; ns, not significant.

FoxO proteins function as important effector arms of the PI3K‐AKT signaling axis by nuclear–cytoplasmic shuttling and driving transcription of apoptotic genes, thus contributing to cellular proliferation and survival.^[^ ^37^ ^]^ The transcriptome data produced by our lab revealed only one *FoxO* gene transcript in *R. dorsalis*. The separation of nuclear and cytoplasm of ovaries from V^+^ and V^−^ females demonstrated increased nuclear FoxO and decreased cytoplasmic FoxO in ovaries of V^+^ females relative to those of V^−^ females (**Figure** **5**A). This suggests that RGDV infection likely induces FoxO translocation from the cytoplasm to the nucleus to enhance the transcriptional activity of *FoxO*.

Conventionally, FoxO mediates mitochondria‐dependent apoptosis by driving transcription of Bcl‐2 family members, which predominantly localize to mitochondria.^[^ ^38^ ^]^ To identify which leafhopper Bcl‐2 family genes have transcription regulated by FoxO, *FoxO* expression in V^−^ females was knocked down. Knockdown of *FoxO* significantly decreased the expression levels of *BNIP3*, *Bax*, and *Bcl‐2‐related Ovarian Killer* (*BOK*) genes (GenBank accession PV492207.1) (Figure 5B). Yeast one‐hybrid assays indicated that FoxO specifically bound to the 5′ non‐coding region (‐2000/‐1 bp) of *BOK*, but not to those of *BNIP3* or *Bax* (Figure 5C). The *BOK* gene encodes a protein containing 276 amino acids and processing domains of BH1 to BH4, which are characteristics of the Bcl‐2 family (Figure S3A, Supporting Information).^[^ ^39^ ^]^ As a pro‐apoptosis member of the Bcl‐2 family, the precise role of BOK in apoptosis regulation has previously been unclear and controversial.^[^ ^39^, ^40^, ^41^ ^]^ Importantly, BOK specifically accumulated in leafhopper ovaries (Figure S3B, Supporting Information), making it a prime candidate for further investigation.

The 5′ non‐coding region of *BOK* was predicted to contain four potential FoxO binding regions, including regions A (‐1829/‐1819 bp), B (‐858/‐849 bp), C (‐629/‐619 bp), and D (‐132/‐121 bp) (Figure S3A, Supporting Information). Preliminary electrophoretic mobility shift assays (EMSAs) revealed a shifted band caused by the incubation of His‐FoxO with the biotin‐labeled probe specific for region B (Figure S3C, Supporting Information). Further EMSAs showed that the presence of antibodies against FoxO in the incubation of His‐FoxO with the probe caused the generation of a super‐shift band and a shift band (Figure 5D). With an increase in the level of the competitor, which was the unlabeled probe and also specific for region B, the binding of FoxO to the probe was reduced, indicating the affinity between FoxO and the probe (Figure 5D). Dual‐luciferase assays demonstrated an increase in the luciferase to *Renilla* luciferase (LUC/REN) ratio in cells expressing FoxO and containing region B (Figure 5E). These results indicate that FoxO could activate the transcription of *BOK* in vitro by binding to region B. The chromatin immunoprecipitation (ChIP) assays in V^+^ females showed specific amplification of region B using corresponding primer sets (Figure 5F). Thus, region B was highly enriched in the chromatin immunoprecipitated with FoxO antibodies. These data demonstrate that FoxO drives *BOK* transcription, with region B at −838–−849 bp in the 5′ terminus of *BOK* serving as the core FoxO binding site.

To confirm the biological function of FoxO in regulating *BOK* transcription, *FoxO* was knocked down in V^+^ females. Northern blot and western blot assays showed a decrease in the accumulation of mRNA and protein of BOK in ovaries caused by knocking down *FoxO* (Figure 5G,H). These results confirm that FoxO drives *BOK* transcription. Combined with the upregulation of FoxO by RGDV, these results illustrate that RGDV infection promotes FoxO to drive *BOK* transcription, thus inducing apoptosis in ovaries.

### 2.6 RGDV Stimulates BOK to Regulate Apoptosis of Ovaries and Eggs

Next, the ability of BOK to regulate the apoptosis of ovaries and eggs was examined. Knocking down *BOK* in V^−^ females reduced the expression of *BOK*, *caspase‐2*, *caspase‐8*, and *IAP*, as well as the accumulation of BOK and cleaved caspase‐8 proteins, in ovaries and eggs produced by these females after mating with V^−^ males (Figure S3D,E, Supporting Information). These findings demonstrate that BOK plays a regulatory role in the apoptosis of ovaries and eggs. However, knocking down *BOK* in V^−^ females had a limited effect on the number and development of eggs, including their size, development duration, or hatching rate (Figure S3F, Supporting Information).

The effect of RGDV infection on the BOK expression of the ovaries was then examined. RT‐qPCR, northern blot, and western blot assays showed increases in the expression and transcription of *BOK* and accumulation of BOK protein in ovaries and eggs produced by V^+^ virgin female × V^−^ male crosses (Figure 5I–K). Knocking down *BOK* in V^+^ females reduced the expression of *BOK*, *caspase‐2*, *caspase‐8*, and *IAP*, the accumulation of BOK and cleaved caspase‐8 proteins, as well as the generation of DNA fragmentation (Figure 5L–N). Furthermore, mitochondrial fractionation revealed that knocking down *BOK* in V^+^ females increased the accumulation of cytochrome C in mitochondria, but it decreased cytochrome C accumulation in the cytosol derived from ovaries, suggesting the reduced release of cytochrome C from mitochondrial to cytosol (Figure 5O). These results indicate that BOK mediates RGDV‐induced mitochondria‐dependent apoptosis in ovaries. The biological assays showed that knocking down *BOK* in V^+^ females also increased the number, hatching rate, and size of eggs produced by these females mating with V^−^ males, while shortening egg development duration (Figure 5P). These data indicate that virus‐induced BOK is also the key factor regulating egg development. Moreover, knocking down *BOK* in V^+^ females significantly decreased the RGDV transovarial transmission rate (Figure 5Q). Taken together, these data indicate that RGDV induces FoxO to drive the transcription of *BOK*, which then triggers the downstream apoptosis cascade of ovaries, thus promoting maternal transmission of RGDV.

### 2.7 ILP2 Recognizes RGDV P2 to Trigger PI3K Signaling

**Figure 6:**
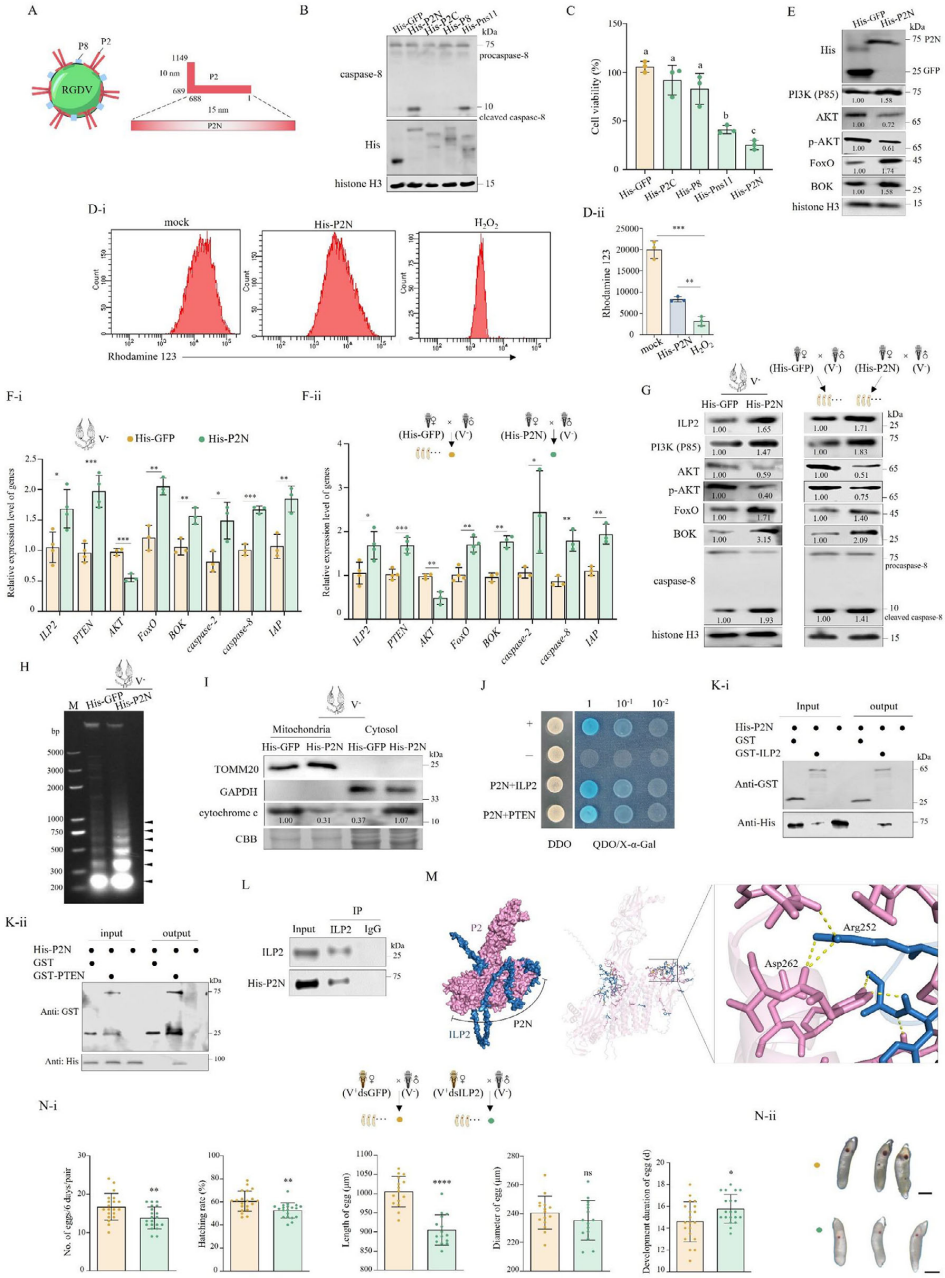
RGDV P2 interacts with ILP2 to trigger ILP‐driven PI3K‐AKT signaling. A) Schematic representation of the structures of a typical RGDV particle and P2 protein. The L‐shaped structure of P2 comprises a 10‐nm domain (amino acids [aa] 1–688, P2N) toward the exterior and a 15‐nm domain (aa 689–1149) on the viral surface. B) P2N expression in Sf9 cells increased the accumulation of cleaved caspase‐8. C) P2N expression in Sf9 cells reduced cellular viability (*n* = 1, three biological replicates). At 48 hpi, Sf9 cells were analyzed with the CCK‐8 assay. The significance of differences was determined using Tukey's honestly significant difference test following one‐way analysis of variance at a *p* < 0.05 threshold. Different letters above columns indicate that the means differ significantly. D) Flow cytometric analysis showing P2N expression reduced mitochondrial membrane potential in Sf9 cells at 48 hpi. Sf9 cells expressed His‐GFP or His‐P2N were stained with rhodamine 123. Means (±SD) in D‐ii show the intensity of rhodamine 123 staining (*n* = 1, three biological replicates). E) P2N expression in Sf9 cells triggered but reversed the PI3K/AKT/FoxO signaling axis (*n* = 1, three biological replicates). F, G) Microinjection with P2N proteins into females triggered but reversed the PI3K/AKT/FoxO signaling axis in ovaries and eggs (*n*_ovary_ = 30, *n*_egg_ = all eggs produced by four pairs of each mating combination, at least three biological replicates in F; *n*_ovary_ = 20, *n*_egg_ = all eggs produced by 12 pairs of each mating combination, three biological replicates in G). H) Microinjection with P2N proteins into females induced chromosomal DNA fragmentation of ovaries (*n*_ovary_ = 40, *n*_egg_ = all eggs produced by five pairs of each mating combination, three biological replicates). Lane M, DNA marker. I) Microinjection with P2N proteins into females induced cytochrome C translocation from mitochondria to cytosol fractions of ovaries (*n*_ovary_ = 30, three biological replicates). Cytosol marker protein GAPDH and mitochondrial marker protein TOMM20 served as fraction controls. J) Y2H assays showing the interaction of P2N with ILP2 and PTEN. Transformants are labeled as follows: P2N+ILP2, pGADT7‐P2N/pGBKT7‐ILP2; P2N+PTEN, pGADT7‐P2N/pGBKT7‐PTEN; +, positive control, i.e., pGBKT7‐53/pGADT7‐T; ‐, negative control, i.e., pGBKT7‐Lam/pGADT7‐T. DDO, SD/‐Trp‐Leu medium; QDO, SD/‐Trp‐Leu‐His‐Ade medium. K) GST pull‐down assay showing the interaction of P2N with ILP2 and PTEN. L) Co‐IP assays showing microinjected His‐P2N interacting with ILP2 of leafhoppers (*n* = 100, three biological replicates). M) Structure of the P2‐ILP2 complex predicted by the AlphaFold 3. In the visual representation, P2 is depicted in pink, whereas ILP2 is depicted in blue. N) Knocking down *ILP2* in V^+^ females reduced progeny egg number, hatching rate, and size and inhibited egg development (*n*_pair_ = 1, at least 15 biological replicates). Bars, 300 µm. The relative intensities of bands for the proteins in E, G, and I are shown below the corresponding bands. Bands of histone H3 indicate the loading of equal amounts of protein in E and G. Statistical analyses of D‐ii, F, and N‐ii were performed using Two‐tailed Student's *t*‐tests. Error bars show the SD. ^*^, *p* < 0.05; ^**^, *p* < 0.01; ^***^, *p* < 0.001; ^****^, *p* < 0.0001; ns, not significant.

To understand how RGDV induced apoptosis of ovaries via triggering but reversing the ILP‐driven PI3K/AKT/FoxO signaling axis, the viral capsid proteins P2 and P8, as well as the nonstructural protein Pns11, were individually expressed in *Spodoptera frugiperda* (Sf9) cells. The expression of the N terminus of P2 (P2N) with a 15‐nm domain, which was oriented toward the exterior of the vririon (**Figure** **6**A), and Pns11 in Sf9 cells caused the accumulation of cleaved caspase‐8, compared to expression of the C terminus of P2 (P2C), with a 10‐nm‐long domain and P8 (Figure 6B). Cell Counting Kit‐8 (CCK‐8) assay showed the lowest cell viability of P2N‐expressing Sf9 cells (Figure 6C). Thus, P2N was determined to likely induce the observed apoptosis. Then, P2N was selected for further investigation.

To determine whether P2N alone disrupted mitochondrial membrane potential, flow cytometry was performed on cells stained with rhodamine 123, a mitochondria‐specific fluorescent dye. P2N expression significantly decreased mitochondrial membrane potential of Sf9 cells (Figure 6D), indicating induction of mitochondria‐dependent apoptosis. More importantly, the expression of P2N in Sf9 cells also increased the accumulation of P85, FoxO, and BOK proteins, but reduced the accumulation of AKT and p‐AKT proteins (Figure 6E). These findings suggest that P2N probably induces mitochondria‐dependent apoptosis of Sf9 via inducing PI3K but inhibiting the AKT/FoxO signaling axis, as observed in RGDV infection.

To confirm the role of P2N in the apoptosis of ovaries, purified P2N that was prokaryotically expressed in *E. coli* strain BL21 was microinjected into female leafhoppers. Microinjection of P2N increased the expression *ILP2*, *FoxO*, *BOK*, *caspase‐2*, *caspase‐8*, and *IAP* genes, enhanced the accumulation of P85, FoxO, BOK, and cleaved caspase‐8 proteins in ovaries and eggs, but decreased the accumulation of AKT and p‐AKT proteins (Figure 6F,G). Injection with P2N also induced DNA fragmentation of ovaries and promoted the translocation of cytochrome C from mitochondria to the cytosol of ovaries (Figure 6H,I). These results reveal that P2 is the key viral protein that induces the reversal of the ILP‐driven PI3K/AKT/FoxO signaling axis, which then triggers mitochondria‐dependent apoptosis of female ovaries.

To determine how P2 reversed the ILP‐driven PI3K/AKT/FoxO signaling axis, the interaction of components in this axis with P2N was examined. P2 was determined to interact with ILP2 (GenBank accession PV492206.1) and PTEN (GenBank accession PV492205.1) based on results of the yeast two‐hybrid (Y2H) system and glutathione *S*‐transferase (GST) pull‐down assays (Figure 6J,K). Co‐immunoprecipitation (Co‐IP) assays further confirmed that the purified P2N that was microinjected into females possessed a high affinity to ILP2 (Figure 6L). Molecular docking with AlphaFold 3 predicted that P2 adopts an L‐shaped flexible conformation, enabling specific binding to ILP2 (Figure 6M). ILP2 was also highly expressed in the ovaries of V^−^ females (Figure S4, Supporting Information). Knocking down *ILP2* in V^+^ females significantly decreased the number, hatching rate, and size of eggs, while prolonging the development of eggs (Figure 6N). Combined with the results indicating that ILP2 functioned in RGDV‐induced ILP‐driven signaling (Figure 4), these findings indicate that ILP2 detects RGDV P2 through its interaction to trigger ILP‐driven PI3K signaling.

### 2.8 RGDV P2 Interacts with PTEN to Reverse the AKT/FoxO Signaling Axis

PTEN is a phosphatase that inhibits the PI3K/AKT signaling pathway by converting PIP3 to PIP2, thus suppressing cell growth, proliferation, and survival in mammals.^[^ ^42^ ^]^ Phylogenetic analysis based on inferred amino acid sequences showed that PTEN of *R. dorsalis* is closely related to PTEN from other insect species in the order Hemiptera (Figure S5A, Supporting Information). Knocking down *PTEN* in V^−^ females reduced the expression of *PTEN*, *FoxO*, *BOK*, *caspase‐2*, *caspase‐8*, and *IAP* genes, as well as the accumulation of BOK and cleaved caspase‐8 proteins in ovaries and eggs produced by these females after mating with V^−^ males; however, its knockdown also increased AKT expression and p‐AKT accumulation (Figure S5B,C, Supporting Information). These results reveal that leafhopper PTEN protein negatively regulates the AKT/FoxO axis and promotes apoptosis of ovaries and eggs.

**Figure 7:**
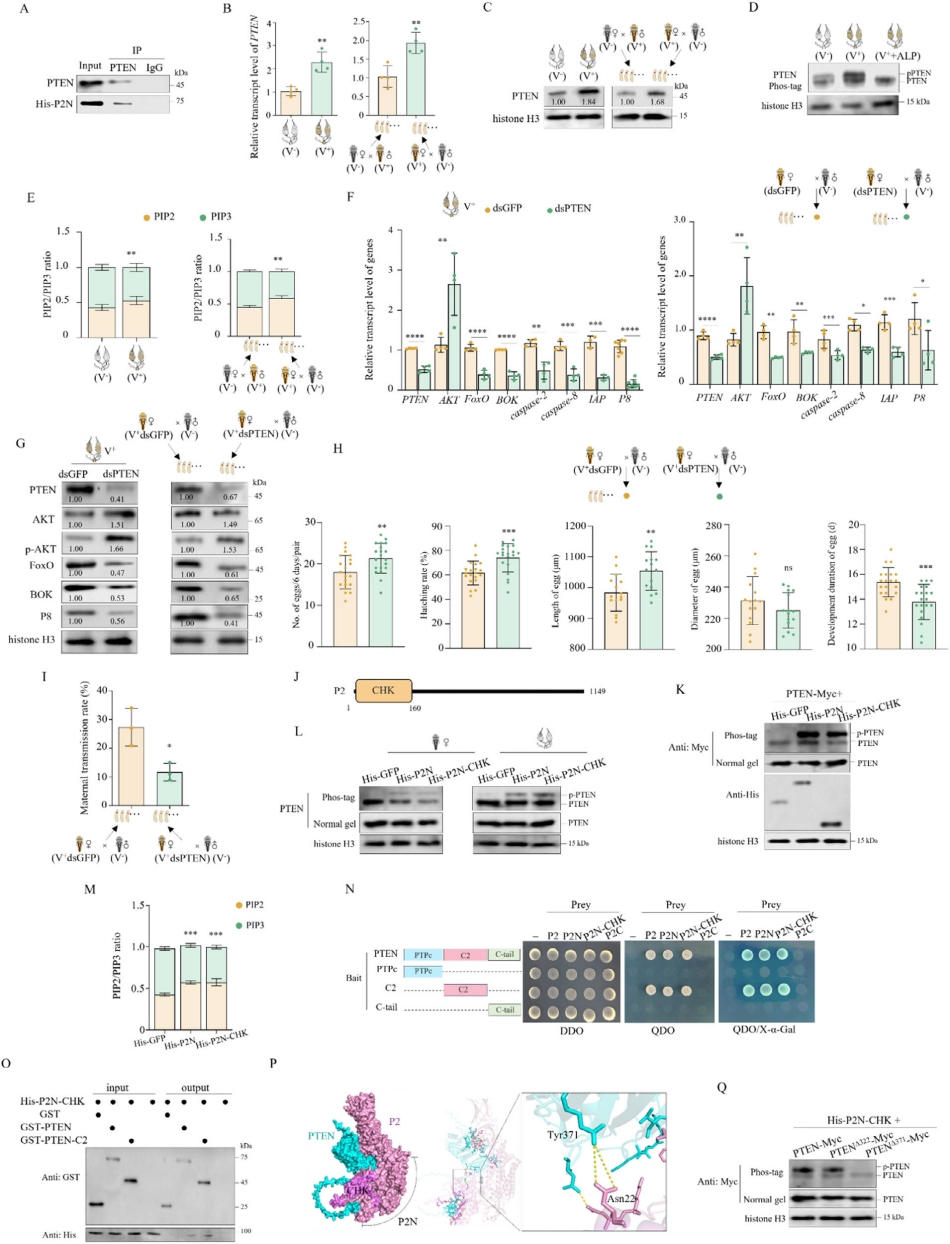
RGDV P2 interacts with PTEN to inhibit the AKT/FoxO signaling axis. A) Co‐IP assays showing the interaction of microinjected His‐P2N with PTEN in leafhoppers (*n* = 100, three biological replicates). B) RGDV infection of females increased expression of *PTEN* in ovaries and eggs (*n*_ovary_ = 30, *n*_egg_ = all eggs produced by four pairs of each mating combination, four biological replicates). C) RGDV infection of females increased accumulation of PTEN in ovaries and eggs (*n*_ovary_ = 20, *n*_egg_ = all eggs produced by 12 pairs of each mating combination, three biological replicates). D) RGDV infection of females increased accumulation of phosphorylated PTEN in ovaries (*n*_ovary_ = 30, three biological replicates). Alkaline phosphatase (ALP) treatment served as the negative control. E) RGDV infection of females increased the PIP2/PIP3 ratio in ovaries and eggs (*n*_ovary_ = 30, *n*_egg_ = all eggs produced by five pairs of each mating combination, with three biological replicates). F, G) Knocking down *PTEN* in V^+^ females promoted the AKT/FoxO signaling axis, but inhibited downstream apoptosis and RGDV infection in ovaries and eggs (*n*_ovary_ = 30, *n*_egg_ = all eggs produced by four pairs of each mating combination, four biological replicates in F; *n*_ovary_ = 20, *n*_egg_ = all eggs produced by 12 pairs of each mating combination, three biological replicates in G). H) Knocking down *PTEN* in V^+^ females increased egg number, hatching rate, and size, and shortened the duration of egg development (*n*_pair_ = 1, at least 15 biological replicates). I) Knocking down *PTEN* in V^+^ females reduced RGDV maternal transmission (*n*_pair_ = 1, four biological replicates). J) Schematic illustration describing the conserved domain of P2. K) Co‐expression of PTEN‐Myc and P2N or P2N‐CHK in Sf9 cells increased the accumulation of phosphorylated PTEN‐Myc. L) Microinjection with P2N‐ or P2N‐CHK‐Myc increased the accumulation of phosphorylated PTEN in insect bodies and ovaries of females (*n*_females_ = 10, *n*_ovary_ = 30, three biological replicates of each). M) Microinjection with P2N‐ or P2N‐CHK‐Myc increased the PIP2/PIP3 ratio in ovaries (*n* = 15 females, three biological replicates). N) Y2H assays showing the interaction of P2N‐CHK with PTEN‐C2. Transformants are labeled as follows: P2N, pGADT7‐P2N; P2N‐CHK, pGADT7‐P2N‐CHK; P2C, pGADT7‐P2C; Bait, pGBKT7‐; +, positive control, i.e., pGBKT7‐53/pGADT7‐T; ‐, negative control, i.e., pGBKT7‐Lam/pGADT7‐T. DDO, SD/‐Trp‐Leu medium; QDO, SD/‐Trp‐Leu‐His‐Ade medium. O) GST pull‐down assay showing the interaction of P2N‐CHK with PTEN‐C2. The bait was PTEN or PTEN‐C2 fused with GST‐tag, the control was GST‐tag, and P2N or P2N‐CHK fused with His was the prey. P) Structure of the P2‐PTEN complex predicted by the AlphaFold 3. In the visual representation, P2 is depicted in pink, whereas PTEN is depicted in cyan. Q) Co‐expression of His‐CHK and PTENΔ322 promoted the accumulation of phosphorylated PTEN‐Myc in Sf9 cells. The relative intensities of bands for the proteins in C and G are shown below the corresponding bands. Bands of histone H3 indicate the loading of equal amounts of protein. Statistical analyses of B, E, F, H, I, and M were performed using Two‐tailed Student's *t*‐tests. Error bars show the SD. ^*^, *p* < 0.05; ^**^, *p* < 0.01; ^***^, *p* < 0.001; ^****^, *p* < 0.0001; ns, not significant.

The interaction of P2N with PTEN in vitro (Figure 6J,K)suggested that PTEN was likely to be the key factor reversing AKT/FoxO signaling axis during RGDV infection. Co‐IP assays showed that the purified P2N that was microinjected into females possessed a high affinity to ILP2 (**Figure** **7**A). RGDV infection significantly increased PTEN expression in ovaries and eggs (Figure 7B,C). The phosphorylation test using Phos‐tag SDS‐PAGE showed increased accumulation of phosphorylated PTEN (p‐PTEN) after RGDV infection (Figure 7D), indicating improved phosphatase activity. The increased PIP2/ PIP3 ratio in the ovaries of V^+^ females and eggs produced by V^+^ virgin female × V^−^ male crosses (Figure 7E) indicated that RGDV‐induced phosphatase activity of PTEN dephosphorylates PIP3 to PIP2. Knocking down *PTEN* in V^+^ females decreased expression of *PTEN*, *FoxO*, *BOK*, *caspase‐2*, *caspase‐8*, *IAP*, and *P8* genes and the accumulation of PTEN, FoxO, BOK, and P8 proteins in ovaries and eggs produced by these females after mating with V^−^ males, but increased the accumulation of AKT or p‐AKT protein (Figure 7F,G). These results indicate that RGDV likely exploits the interaction of P2N with PTEN to increase the accumulation and phosphatase activity of PTEN, thereby inhibiting the PI3K/AKT signaling pathway and thus facilitating infection. Knocking down *PTEN* in V^+^ females also increased the number, hatching rate, and size of eggs, but shortened the development duration of these eggs and suppressed RGDV maternal transmission (Figure 7H,I). Collectively, these data indicate that P2N interaction with PTEN inhibits the AKT/FoxO signaling axis, thus promoting downstream apoptosis and maternal transmission.

We further investigated how the phosphatase activity of PTEN was enhanced by the interaction of P2N with PTEN. The inferred amino acid sequence of P2N contained a choline kinase (CHK) domain from position 1–160, referred to as P2N‐CHK (Figure 7J), suggesting its potential ability for phosphorylation. Co‐expression of PTEN with P2N or P2N‐CHK in Sf9 cells promoted the accumulation of p‐PTEN, indicating that P2N‐CHK improved phosphatase activity of PTEN in Sf9 cells (Figure 7K). Microinjection of females with purified P2N or P2N‐CHK specifically increased the accumulation of p‐PTEN in both the ovaries and the insect bodies, as well as the ratio of PIP2 to PIP3 (Figure 7L,M). These findings suggest that P2N‐CHK is likely to be the key domain phosphorylating PTEN, thus increasing the phosphatase activity of PTEN.

The amino acid sequence of PTEN contained several domains, i.e., a protein tyrosine phosphatase domain, a catalytic domain (PTPc), a C2 domain (referred to as PTEN‐C2), and a C tail domain (Figure 7N). Y2H and GST pull‐down assays showed that P2N‐CHK interacted with PTEN‐C2 (Figure 7N,O), suggesting that PTEN‐C2 and P2N‐CHK are likely to be the key domains underlying the interaction of PTEN with P2. It has been reported that phosphorylation of the C2 domain can increase the phosphatase activity of PTEN in human cells.^[^ ^43^ ^]^ PTEN‐C2 of *R. dorsalis* contained two tyrosine residues, which are susceptible to phosphorylation,^[^ ^43^ ^]^ at amino acid residues 322 and 371. Molecular docking analysis using AlphaFold 3 revealed that the tyrosine residue at position 371 in PTEN was in closest proximity to P2 (Figure 7P), suggesting its potential role as a critical binding site mediating the P2‐PTEN interaction. Then tyrosine mutants at positions 322 and 371 were constructed, referred to as PTENΔ322 and PTENΔ371, respectively. Co‐expression of P2N‐CHK and PTENΔ371 in Sf9 cells failed to promote the accumulation of p‐PTEN, while the co‐expression of P2N‐CHK and PTENΔ322 still promoted p‐PTEN accumulation (Figure 7Q). These results indicate that the tyrosine at position 371 of PTEN is the likely key phosphorylation site targeted by P2‐CHK.

### 2.9 Other Rice Viruses Exclusively Transmitted Maternally do not Induce Apoptosis in Ovaries

To determine whether other rice viruses that were transmitted exclusively through females also induce ovarian apoptosis in their insect vectors, rice dwarf virus (RDV), transmitted by the leafhopper *Nephotettix cincticeps*, and RSV, transmitted by the small brown planthopper *Laodelphax striatellus* Fallén, were investigated. Both viruses are vertically transmitted only through females. Infection of these viruses had a limited effect on the accumulation of cleaved caspase‐8 in ovaries, though RSV infection increased caspase‐3 activity in ovaries of planthoppers (Figure S6A,B, Supporting Information). Moreover, infection did not cause apoptotic DNA fragmentation in ovaries (Figure S6C, Supporting Information). RDV infection did not impair ovarian development, while RSV infection notably inhibited ovary development (Figure S6D, Supporting Information), consistent with previous findings.^[^ ^44^ ^]^ These data indicate that other rice viruses exclusively relying on maternal transmitted do not induce ovarian apoptosis. In contrast, RGDV, which exploits maternal transmission as a supplementary means of transmission, specifically induces apoptosis of ovaries and eggs to promote infection but imposes a fitness cost on female fecundity.

## 3 Discussion

Investigation of the co‐evolution of pathogens and insect vectors has been an active and challenging research field, and the insect fitness costs imposed by pathogens are among the most important biotic sources of selective pressure in these systems. Some insect vectors show reduced fitness under persistent‐propagative transmission of plant viruses, especially in the form of impaired growth and development of insect vectors or male tissue differentiation in genetic females.^[^ ^4^, ^5^, ^7^, ^8^, ^9^ ^]^ In this study, the mechanism by which a reovirus that is vertically transmitted in a dominant‐paternal and supplementary‐maternal manner reduces female reproduction fitness has been revealed to involve apoptosis.

Our previous work established that RGDV induces mitochondria‐mediated apoptosis to facilitate viral infection in cultured cells of leafhoppers.^[^ ^33^ ^]^ Multiple approaches, including electron microscopy, immunofluorescence, flow cytometry, and DNA fragmentation assays, were used to systematically examine cytopathological changes and biochemical markers of apoptosis. These in vitro findings were further validated in whole insects and dissected guts, combining ultrastructural analysis, immunofluorescence of gut epithelial cells, and quantitative viral titer measurements under both infection and RNAi conditions.^[^ ^33^ ^]^ Building on these results, the current study shows that RGDV specifically induces mitochondria‐dependent apoptosis in ovaries. Because RGDV infection of the ovaries is slow and inefficient,^[^ ^30^ ^]^ once the virus enters, it induces apoptosis, thus promoting infection to compensate for the loss of previous viral particles being blocked by the ovary infection barrier. At the same time, however, RGDV infection of the ovaries also impairs female reproduction. This apoptosis can also be transgenerationally inherited by offspring, further affecting egg development. At the molecular level, RGDV infection of ovaries activates the ILP/PI3K signaling pathway but reverses the downstream AKT/FoxO signaling axis, ultimately inducing apoptosis in a way that benefits viral infection. These results reveal that the fitness cost to females and offspring caused by the maternal transmission of RGDV limits the growth of the vector population. In contrast, these responses were observed to be absent during paternal transmission of RGDV. The transcription of ovary‐specific BOK driven by the AKT/FoxO signaling axis enables RGDV to specifically induce apoptosis, thus impairing female reproduction and degenerating egg development, but without affecting male reproduction. Therefore, females exhibit maladaptation to the vertical transmission of RGDV.

These findings provide support for the virulence‐transmission trade‐off hypothesis,^[^ ^45^, ^46^, ^47^ ^]^ which suggests that successful pathogens must balance transmission efficiency with host fitness costs to maximize reproductive potential. In this system, we observed two distinct evolutionary strategies with contrasting outcomes. Maternal transmission ensures vertical transmission passage but carries evolutionary limitations. First, virus‐induced cytopathology in oocytes directly impairs offspring viability. Second, the reduced lifespan of infected females decreases the transmission efficiency. When these costs outweigh the benefits, maternal transmission is subject to negative selection, consistent with the relatively low field‐recorded transmission rate (≈20%).^[^ ^28^ ^]^ In contrast, paternal transmission achieves higher efficiency (≈60%)^[^ ^28^ ^]^ without impairing offspring development, providing a clear evolutionary advantage. The coexistence of these two transmission modes likely reflects viral adaptation to different selective pressures. During challenging periods such as winter, maternal transmission alone may be insufficient for persistence, prompting RGDV to evolve a complementary paternal transmission strategy that has successfully maintained its endemic status in southern China for over four decades. This biparental transmission mode provides a more favorable balance between viral spread and insect fitness.

Insect innate antiviral immunity typically involves RNAi, autophagy, and signaling pathways such as Toll, IMD, and JAK‐STAT.^[^ ^48^, ^49^ ^]^ Because insect eggs at the embryonic stage lack differentiated immunity, some immune responses can be inherited from parents to offspring to execute functions.^[^ ^50^, ^51^, ^52^, ^53^, ^54^, ^55^, ^56^ ^]^ This study provides evidence for transgenerational apoptosis, indicated by mitochondrial pathway activation and correlations with viral load in offspring. The molecular basis of this inheritance will be a subject of future research.

Apoptosis generally functions as an immunity response to limit viral infection. Apoptosis of insect vectors inhibits infection by baculoviruses or transmission of arboviruses, including West Nile virus, Dengue virus‐2, and Zika virus.^[^ ^17^, ^18^, ^57^ ^]^ Viruses also utilize apoptosis to facilitate infection and transmission, as observed in African swine fever virus, Ascovirus, and Begomovirus.^[^ ^58^, ^59^, ^60^, ^61^, ^62^ ^]^ Our findings reveal that RGDV‐induced apoptosis represents a novel variation of this strategy, where the transgenerational inheritance of apoptotic effects supports persistent infection while regulating vector population dynamics through egg degeneration, thereby regulating the long‐term transmission and spread of virus in the ecosystem.

Mitochondria are a cellular hub of immune response against pathogens. Pathogens exploit these roles of mitochondria by affecting mitochondrial networks and disrupting communication between the nucleus and the mitochondria to promote infection.^[^ ^63^ ^]^ For example, RSV infection induces vacuolated mitochondria and irregular cristae observed during apoptosis in the midgut of planthopper vectors.^[^ ^64^ ^]^ Our previous study revealed the close association of RGDV with the mitochondria of its leafhopper vector; in the alimentary canal of leafhoppers, RGDV degenerates mitochondria, leading to mitochondria‐dependent apoptosis.^[^ ^33^ ^]^ To maintain mitochondrial homeostasis, these impaired mitochondria are also recruited into phagophores to initiate mitophagy, which suppresses extensive apoptosis and mitigates virus‐induced fitness costs in vectors.^[^ ^65^, ^66^ ^]^ However, how such mitochondrial damage initiates the downstream apoptosis cascade remained unclear. Bcl‐2 family proteins are localized to the mitochondrial membrane and control mitochondria‐dependent apoptosis. The Bcl‐2 family includes BOK, but whether and how BOK precisely regulates apoptosis has been unclear and controversial previously.^[^ ^39^, ^40^, ^41^ ^]^ Here, we found that BOK, which has transcription driven by FoxO, induces apoptosis in ovaries and therefore promotes the maternal transmission of the virus. In the ovaries of other insect species, BOK may play similar roles in regulating apoptosis.

ILPs of invertebrates are known to be particularly important reproductive regulation factors in addition to hormones and juvenile hormone.^[^ ^67^ ^]^ Interfering with the insulin signaling pathway by knocking down *InR* impairs oocyte maturation.^[^ ^68^ ^]^ Beyond reproduction, ILPs function as an important extracellular signal, and they regulate cellular growth and metabolism and nutritional conditions through PI3K/AKT activation.^[^ ^65^, ^66^, ^69^ ^]^ However, the connection between apoptosis and reproductive development, as mediated by the ILP‐driven PI3K/AKT/FoxO axis, has not been characterized. In the present system, the ILP‐driven PI3K/Akt/FoxO axis is associated with reproductive development of leafhoppers. We found that RGDV interferes with the ILP‐driven PI3K/Akt/FoxO axis of leafhoppers, ultimately activating apoptosis in ovaries. ILP2 in leafhopper is the key extracellular signaling factor that initiates apoptosis in V^+^ ovaries. ILP2 detects viral infection via binding to the RGDV capsid protein P2 and then reverses the PI3K/Akt/FoxO signaling axis, which would otherwise have inhibited the apoptosis cascade and therefore blocked viral infection. To ensure the effective infection of ovaries, RGDV exploits the interaction of P2 with PTEN to inhibit the Akt/FoxO signaling axis. This strategy disturbs the signaling pathway, thus activating apoptosis and promoting viral infection in the ovaries, while simultaneously impairing ovary development and degenerating egg development, which ultimately reduces female fecundity.

Pathogenic functions of RGDV proteins have been reported previously, especially the roles of P2 and Pns11 in inducing the immune response of leafhopper vectors.^[^ ^33^, ^70^, ^71^ ^]^ Both P2 and Pns11 can induce incomplete autophagy to facilitate persistent infection of the leafhopper alimentary canal.^[^ ^70^, ^71^, ^72^ ^]^ Pns11 also acts as a key cytotoxic protein that targets mitochondria and activates apoptosis in the alimentary canal of the leafhopper.^[^ ^33^ ^]^ In the present work, P2 similarly exhibited cytotoxicity in Sf9 cells and individually caused mitochondria‐dependent apoptosis in ovaries. The P2N fragment, exposed on the virion surface, exhibited dual regulatory activity in apoptosis. Extracellularly, ILP2 specifically recognized and bound to P2N, which enhanced ILP2 accumulation and increased its binding affinity for the InR on the cell membrane. This interaction activated the ILP/PI3K signaling pathway. After viral entry, the CHK domain of P2 interacted with and phosphorylated PTEN, enhancing PTEN phosphatase activity. Activated PTEN dephosphorylated PIP3 to PIP2, suppressing PIP3‐dependent AKT activation. Consequently, this suppression reversed the AKT/FoxO signaling axis, ultimately triggering apoptosis.

**Figure 8:**
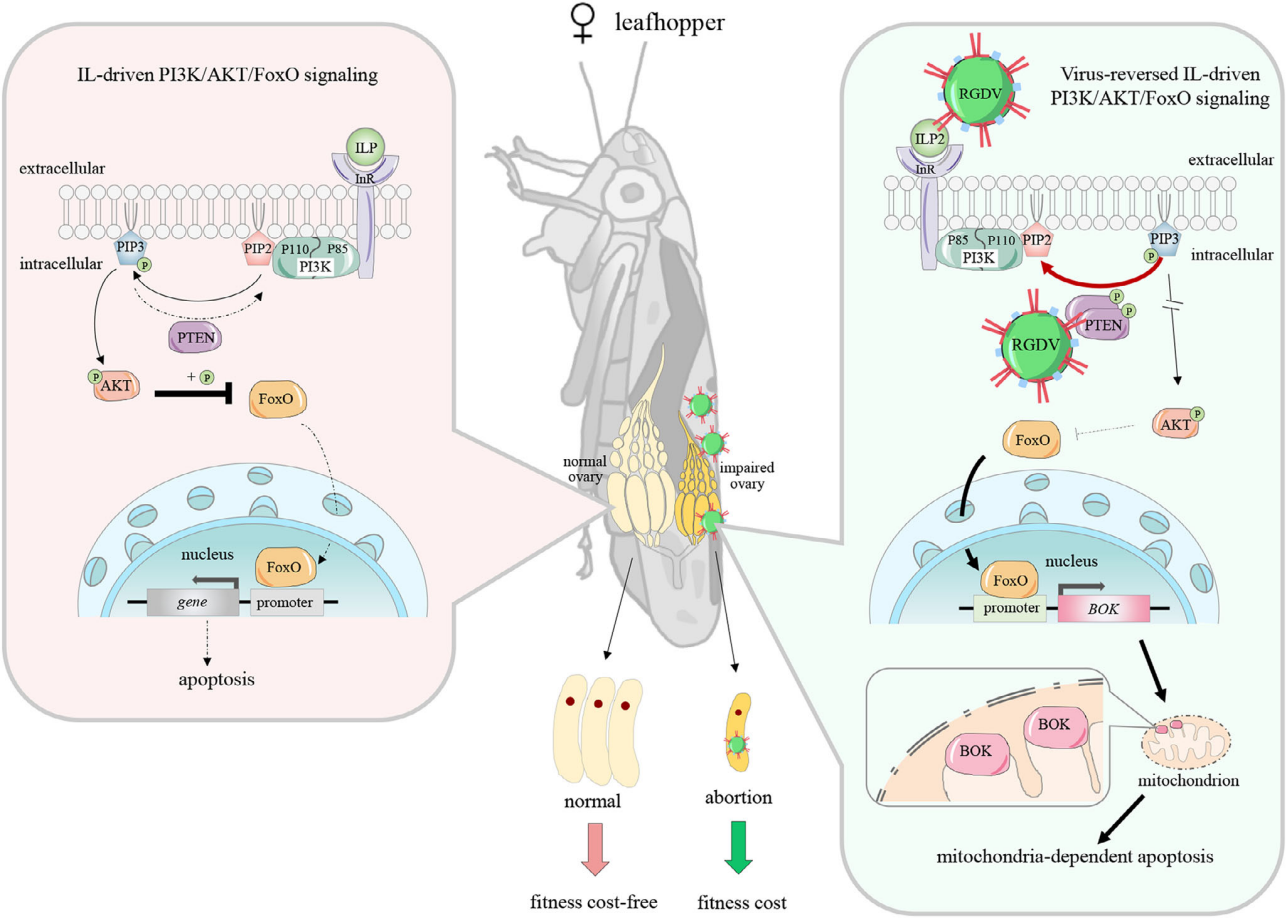
Proposed model of RGDV‐induced apoptosis of the ovary via the ILP‐driven PI3K/AKT/FoxO axis, promoting viral infection but reducing female fitness. When RGDV infects the ovaries of leafhopper, extracellular ILP2 detects capsid protein P2 of RGDV via interaction. The activation of InR by ILP2 induces PI3K to trigger the AKT/FoxO signaling axis, which would otherwise have suppressed downstream apoptosis and therefore blocked viral infection. To continuously infect ovary tissue, RGDV utilizes P2 to increase the accumulation and phosphatase activity of PTEN, thus reversing the production of PIP3 from PIP2, leading to the inhibition of AKT activity. The inactivated AKT allows nuclear export of FoxO from the cytoplasm, thus driving the transcription of *BOK* and ultimately launching the mitochondria‐dependent apoptosis cascade. This induced apoptosis facilitates RGDV infection in ovaries, can even be transgenerationally inherited by eggs, further promoting viral infection. However, this induced apoptosis impairs ovary development and causes degenerated egg growth, ultimately imposing a fitness cost on females.

Based on these findings, we propose a novel model of the conflict between the virus infection and the ILP‐driven PI3K/AKT/FoxO axis in the ovary of leafhoppers (**Figure** **8**). Extracellular ILP2 detects RGDV infection via its binding to RGDV capsid protein P2 and then activates InR to trigger the PI3K/AKT/FoxO signaling axis, which would otherwise inhibit the apoptosis cascade and therefore block viral infection in ovaries. To effectively infect ovaries, RGDV exploits P2 to increase the accumulation and phosphatase activity of PTEN, thus preventing PIP3 accumulation, suppressing AKT, and promoting FoxO‐mediated BOK transcription. This cascade ultimately triggers mitochondria‐dependent apoptosis, which can be transgenerationally inherited to eggs, thus promoting viral infections in both the ovaries and eggs. However, RGDV infection‐associated apoptosis simultaneously impairs ovary development and degenerates egg growth, ultimately reducing female reproductive fitness.

## 4 Experimental Section

### Insects, Viruses, and Antibodies

V^−^ *R. dorsalis* leafhoppers were originally obtained from Fujian Province in southern China and were subsequently propagated for several generations at 25 ± 3 °C in a greenhouse at Fujian Agriculture and Forestry University. All rice plants used in this study belonged to the *Oryza sativa* L. ssp. *japonica* cultivar “Nipponbare.” The rice plants were cultivated in a greenhouse maintained at 28–32 °C, with a relative humidity of 60% ± 5% and natural sunlight. RGDV‐infected rice plants were collected from Fujian Province, and the virus was propagated to other rice plants through transmission by *R. dorsalis* under greenhouse conditions.

Polyclonal rabbit antibodies against caspase‐8, RGDV P8, TOMM20, GAPDH, ILP2, InR2, P85, AKT, pAKT, BOK, PTEN, FoxO, and Cytochrome C antigens were prepared by Genscript Biotech Corporation (Nanjing, China). The procedures were approved by the Science and Technology Department of Jiangsu Province, China. Mouse monoclonal antibodies against 6×His‐tag and GST were purchased from TransGen Biotech (Beijing, China; HT501, HT601). Goat anti‐rabbit IgG‐peroxidase or goat anti‐mouse IgG‐peroxidase was used as the secondary antibody (Sangon, Shanghai, China; D110058 or D110087, respectively). Alexa Fluor 647 Phalloidin was procured from Thermo Fisher Scientific (A22287). IgGs against RGDV antigen were directly conjugated to rhodamine following the manufacturer's instructions (Thermo Fisher Scientific, USA).

### Viral Acquisition by Insects

The second instar V^−^ nymphs were fed on RGDV‐infected rice plants for 3 d and then were transferred to RGDV‐free rice seedlings. The preliminary RT‐PCR assays showed that this manner of viral acquisition caused an 80% infection rate in the newly emerged population after a latent period of 14 d. By 14 d post first access to diseased plants (padp), most leafhoppers had reached the adult stage.

At 3, 6, 9, or 12 d after eclosion, ovaries or testes were dissected from the insect bodies. These bodies were also individually tested for the presence of *P8* gene transcripts using RT‐PCR assays. Then, ovaries or testes of individuals that were positive for *P8* were analyzed in the assays described below.

### Immunofluorescence Microscopy

At 9 d after eclosion, dissected ovaries were fixed, immunolabeled with viral particles‐specific IgG conjugated to rhodamine (virus‐rhodamine) and phalloidin‐FITC, and processed for immunofluorescence microscopy, as described previously.^[^ ^30^ ^]^ As a control, ovaries from virus‐free females (fed on healthy plants) were treated in the same way.

### Effect of RGDV Infection on Expression of Apoptosis‐, Ecdysone‐, or Juvenile‐Related Factors in Ovary and Testis

At 3, 6, 9, or 12 d after eclosion, ovaries and testes from RGDV‐positive individuals were analyzed for the expression of *caspase‐2*, *caspase‐8*, *IAP*, *EcR*, *E75*, *Kr‐h1*, *Met*, and *P8* using RT‐qPCR assays. Total RNA samples were extracted from the ovaries of 15 female adults for first‐strand cDNA synthesis using M‐MLV Reverse Transcriptase (Promega, Madison, WI, USA). The qPCR was performed using the 2 × Real Star Green Fast Mixture (with ROX II) (GenStar, Shenzhen, China; A303). The *EF‐1α* transcript of *R. dorsalis* served as the internal reference for the normalization of gene expression levels. Relative gene expression levels were calculated using the 2^−ΔΔCT^ method. At least four biological replicates were performed.

At 9 d after eclosion, ovaries and testes of RGDV‐positive individuals were also analyzed for P8 expression and caspase‐8 activation using western blot assays. Total proteins from the ovaries or testes of 10 V^+^ female or male adults were examined using western blot assays for P8 and caspase‐8. Three biological replicates were evaluated. Band intensities of proteins analyzed by western blot assays were quantified with Image J software.

### Effect of RGDV Infection on Caspase‐3 Activity of Testis and Ovary

At 9 d after eclosion, ovaries and testes of RGDV‐positive individuals were analyzed for caspase‐3 activity. In brief, ovaries or testes from 15 V^+^ female or male adults, respectively, were ground and incubated with 100 µL of lysis buffer from the Caspase 3 Activity Assay Kit (Biyotime, Shanghai, China; C1116) and then tested for caspase‐3 activity. The absorbance of samples at 420 nm was measured using the SPARK 10 m Microplate spectrophotometer (TECAN, Grödig, Austria). At least three replicates were evaluated for each sample type.

### Effect of RGDV Infection on Chromosomal DNA Fragmentation of Ovary and Testis

At 9 d after eclosion, ovaries and testes of RGDV‐positive individuals were analyzed for chromosomal DNA fragmentation. In brief, ovaries or testes from 20 V^+^ female or male adults were ground and incubated with 100 µL of lysis buffer from the DNA Ladder Detection Kit (KeyGEN BioTECH, Nanjing, China; KGA1300‐100). Then, chromosomal DNA was extracted according to the manufacturer's instructions and separated on 1.0% agarose gels at 50 V for 40 min.

### Effect of RGDV Infection on Translocation of Mitochondrial Cytochrome C in Ovary and Testis

At 9 d after eclosion, ovaries and testes from RGDV‐positive individuals were analyzed for the accumulation of cytochrome C in mitochondria. Ovaries or testes from 15 V^+^ female or male adults, respectively, were ground and treated to isolate mitochondria from the cytosol using Cell Mitochondria Isolation Kit (Beyotime, Shanghai, China; C3601). The accumulations of cytochrome C in mitochondria and cytosol were analyzed using western blot assays. TOMM20 served as a mitochondrial marker, while GAPDH served as a cytosol marker. Three biological replicates were evaluated.

### Inhibition of Apoptosis during RGDV Infection in Ovary

At 9 d after eclosion, potential V^+^ females were microinjected with 120 nL of Z‐VAD‐FMK (10 µm). At 3 d post‐injection, ovaries were dissected from the female bodies. These bodies were individually assayed for virus infection by RT‐PCR assays. Ovaries from 15 V^+^ female adults were analyzed for the expression of *caspase‐2*, *caspase‐8*, *IAP*, and *P8* genes, the accumulation of cleaved caspase‐8, and caspase‐3 activity using RT‐qPCR, western blot assays, and a Caspase 3 Activity Assay Kit, respectively. At least three biological replicates were evaluated.

### Effect of Knocking Down Caspase‐8 or IAP of RGDV Infection in Ovary and Ovarial Development

A T7 RNA polymerase promoter with the sequence 5′‐ATTCTCTAGAAGCTTAATACGACTCACTATAGGG‐3′ was added to the forward and reverse primers for the *caspase‐8*, *IAP*, and *GFP* genes at the 5′ terminus to amplify regions of ≈1200, 750, and 475 bp, respectively. Subsequently, a T7 RiboMAX Express RNAi System (Promega, P1700) was used for in vitro synthesis of dsRNAs targeting *caspase‐8* (dscaspase‐8), IAP (dsIAP), and GFP (dsGFP).

At 9 d after eclosion, potential V^+^ females were microinjected with dscaspase‐8, dsIAP, and dsGFP (≈60 ng per insect) at the intersegmental region of the thorax using a Nanoject II Auto‐Nanoliter Injector (Spring). These microinjected leafhoppers were then transferred to healthy rice seedlings for recovery. At 5 d post microinjection, the ovaries were dissected from the female bodies. These bodies were individually assayed for virus infection by RT‐PCR assays. Ovaries from 15 V^+^ female adults were analyzed for the expression of *caspase‐2*, *caspase‐8*, *IAP*, and *P8* genes using RT‐PCR assays, the accumulation of cleaved caspase‐8 and P8 using western blot assays, and caspase‐3 activity using a Caspase 3 Activity Assay Kit. At least three biological replicates were evaluated. Ovaries from 15 V^+^ female adults were also evaluated for their development using a light microscope (Leica DM500; Leica, Wetzlar, Germany). The length and diameter of oocytes were obtained using Capture 2.0 software.

### Apoptosis Inducer Effects on RGDV Infection in Ovaries and Mortality of Leafhoppers

At 9 d after eclosion, the V^−^ female adults were microinjected with 120 nL of 10% H_2_O_2_. At 2 d post‐injection, ovaries were dissected from the female bodies. A pool of RNAs derived from the ovaries of 15 V^−^ females was analyzed for the expression of *caspase‐2*, *caspase‐8*, *IAP*, and *P8* genes using RT‐qPCR assays and the accumulation of cleaved caspase‐8 and P8 using western blot assays. Four and three biological replicates were evaluated for RT‐qPCR and western blot assays, respectively. Ovaries from 15 V^−^ female adults were analyzed for caspase‐3 activity at 3 d post‐injection, with four biological replicates. Ovaries from 20 V^−^ female adults were analyzed for chromosomal DNA fragmentation at 3 d post‐injection, with three biological replicates.

To investigate the mortality of these leafhoppers, 30 leafhoppers at 1 d post‐injection were allowed to individually feed on a healthy rice seedling in a glass tube for 5 d. Then, mortality was recorded. Four biological replicates were evaluated.

### Analysis of Apoptosis in Eggs Laid by V+ Female Adults

Fifth instar nymphs, after viral acquisition or collection from a V^−^ population, were reared separately in glass tubes until eclosion prior to mating. The newly emerged females and males were collected to establish two mating combinations: (i) V^+^ virgin female × V^−^ male and (ii) V^−^ virgin female × V^+^ male. In each combination, newly emerged females and males mated one to one in glass tubes containing rice seedlings for 5 d. Then, potential V^+^ males from each tube were collected for virus detection by RT‐PCR assays, and the females were left in each of the tubes to lay eggs for 5 d. Rice seedlings in each tube were renewed daily to avoid viral acquisition by leafhopper vectors from plant hosts. After the 5‐d period for egg laying, potential V^+^ females were collected for virus detection by RT‐PCR assay. At 6 d after the removal of females, 10 intact eggs produced by each pair of each of the two mating combinations were harvested by dissecting rice seedlings. A TaqMan Gene Expression Cells‐to‐CT kit (Thermo Fisher Scientific, Waltham, MA, USA; 4399002) was used to lyse the eggs and prepare the RNAs according to the manufacturer's instructions. A pool of RNAs from 10 V^+^ eggs produced by each pair was analyzed for the expression of *caspase‐2*, *caspase‐8*, *IAP*, and *P8* genes using RT‐qPCR assays. Additionally, 50 eggs produced by five mating pairs of each combination were also analyzed for the accumulation of cleaved caspase‐8 and P8 using western blot assays as well as for caspase‐3 activity using a Caspase 3 Activity Assay Kit. At least three biological replicates were subjected to RT‐qPCR, western blot, and caspase‐3 activity assays.

### Effect of Apoptosis in Eggs on RGDV Infection

Newly emerged potential V^+^ females were microinjected with dscaspase‐8, dsIAP, and dsGFP (≈60 ng per insect). At 5 d post post‐injection, three mating combinations were established: (i) dscaspase‐8‐treated V^+^ female × V^−^ male; (ii) dsIAP‐treated V^+^ female × V^−^ male; and (iii) dsGFP‐treated V^+^ female × V^−^ male. In each combination, virgin females and males mated one to one in glass tubes containing rice seedlings for 3 d. Then, the potential V^+^ females were collected for virus detection by RT‐PCR assay. At 6 d after the removal of females, 10 intact eggs produced by each pair of each of the three mating combinations were harvested. A pool of RNAs derived from 10 V^+^ eggs of each pair was analyzed for the expression of *caspase‐2*, *caspase‐8*, *IAP*, and *P8* genes using RT‐qPCR assays. Additionally, 50 eggs produced by five mating pairs of each combination were analyzed for the accumulation of cleaved caspase‐8 and P8 using western blot assays. At least three biological replicates were subjected to RT‐qPCR and western blot assays.

At 6 d after the removal of females from mating combinations, eggs produced by each pair were harvested and counted to obtain their total number. The development of 15 eggs was analyzed by light microscopy, and the length and diameter of eggs were obtained using Capture 2.0 software. Then, the eggs of each pair were placed on pieces of water‐soaked filter paper and monitored to characterize their development and hatching. One pair per mating combination over 6 d was considered one biological replicates, with at least 15 biological replicates.

### Induction of PI3K‐AKT Signaling in Ovaries by RDGV Infection

A pool of RNAs was isolated from ovaries of 15 V^+^ females or eggs produced by four pairs of the following two mating combinations: (i) V^+^ virgin female × V^−^ male and (ii) V^−^ virgin female × V^+^ male. Then, RT‐qPCR assays were conducted to assay the expression levels of *ILP2*, *InR2*, *PI3K*, *Akt*, *FoxO*, and *BOK* genes. Four biological replicates were assayed. A pool of proteins from eggs of 12 mating pairs or from ovaries of 10 V^+^ females was analyzed for the accumulation of ILP2, P85, AKT, p‐AKT, FoxO, BOK, and P8 proteins using western blot assays, with three biological replicates.

### Effect of Knocking Down Genes of Insulin‐Driven PI3K‐AKT Signaling Pathway on Apoptosis in Ovaries and Eggs

A T7 RNA polymerase promoter was added to the forward and reverse primers for the *ILP2*, *InR2*, *PTEN*, *PI3K*, *AKT*, and *FoxO* genes at their 5′ termini to amplify regions of ≈255, 786, 872, 840, 972, and 745 bp, respectively. The T7 RiboMAX Express RNAi System was utilized for in vitro synthesis of dsRNAs targeting ILP2 (dsILP2), InR2 (dsInR2), PTEN (dsPTEN), PI3K (dsPI3K), AKT (dsAKT), and FoxO (dsFoxO).

The newly emerged potential V^+^ females were microinjected with the dsRNAs described above (≈60 ng per insect). At 5 d post injection, the following seven mating combinations were established as follows: (i) dsILP2‐treated V^+^ female × V^−^ male; (ii) dsInR2‐treated V^+^ female × V^−^ male; (iii) dsPTEN‐treated V^+^ female × V^−^ male; (iv) dsPI3K‐treated V^+^ female × V^−^ male; (v) dsAKT‐treated V^+^ female × V^−^ male; (vi) dsFoxO‐treated V^+^ female × V^−^ male; and (vii) dsGFP‐treated V^+^ female × V^−^ male. In each combination, virgin females and males mated one to one in glass tubes containing rice seedlings for 3 d. Then, the potential V^+^ females were collected for virus detection by RT‐PCR assay. At 6 d after the removal of females, 10 intact eggs produced by each pair of each of the seven mating combinations were harvested.

A pool of RNAs was isolated from ovaries of 15 dsRNA‐treated V^+^ females at 5 d post‐injection or eggs produced by four pairs of each mating combination listed above. Then, RT‐qPCR assays were conducted to assay the expression levels of *ILP2*, *InR2*, *PTEN*, *PI3K*, *AKT*, *FoxO*, *caspase‐2*, *caspase‐8*, *IAP*, *P8*, *BNIP3*, *Bax*, *Bik*, *Bad*, *Bid*, *Bak*, and *BOK* genes. A pool of proteins from ovaries of 10 dsRNA‐treated V^+^ females or eggs produced by 12 pairs from each of the seven mating combinations was analyzed for the expression of ILP2, P85, PTEN, AKT, p‐AKT, FoxO, BOK, caspase‐8, or P8 proteins using western blot assays. At least three biological replicates were subjected to RT‐qPCR and western blot assays.

### FoxO Shuttling and BOK Transcription in Ovaries under RGDV Infection

A pool of proteins isolated from ovaries of 10 V^+^ females was analyzed for FoxO accumulation in cytoplasmic and nuclear fractions using a Nuclear and Cytoplasmic Protein Extraction Kit (Beyotime, P0027). Antibodies against tubulin and histone H3 were respectively used as the cytoplasmic and nuclear markers, respectively.

A pool of RNAs derived from ovaries of 10 V^+^ females was subjected to northern blot assays. A 3′‐biotin‐labeled DNA probe (5′‐CCGCCAACCCTCCGGCTACTGCGAACAGCGACACCACCTT‐3′) of *BOK*, corresponding to 541–580 bp, was synthesized (Tsingke Biotech, Beijing, China). Total RNA (≈5–10 µg) from each treatment was loaded and assayed for the transcript level of *BOK*. The rRNA stained with methylene blue served as a control to confirm loading of equal amounts of RNA in each lane. Three biological replicates were assayed.

### Expression of Bcl‐2 Family Related Genes and BOK under FoxO Knockdown

At 9 d after eclosion, the potential V^+^ females were microinjected with dsFoxO or dsGFP (≈60 ng per insect). At 5 d post microinjection, ovaries were dissected from the females, and these same bodies were individually assayed for virus infection by RT‐PCR. A pool of RNAs derived from ovaries of 15 V^+^ females was analyzed for the expression of *BNIP3*, *Bax*, *Bik*, *Bad*, *Bid*, *Bak*, and *BOK* genes using RT‐qPCR assays. A pool of RNAs from ovaries of 10 RGDV^−^positive females was subjected to northern blot assays. Four and three biological replicates were respectively subjected to RT‐qPCR and northern blot assays, respectively.

### Yeast One‐Hybrid Assay

The 5′ upstream potential promoter regions (‐2000/‐1 bp) of *BOK*, *BNIP*, and *Bax* genes were respectively cloned into pAbAi yeast reporter vectors to create bait plasmids (pAbAi‐pBOK, ‐pBNIP, and ‐pBax). These recombinant plasmids were transformed into yeast strain Y1H, followed by the selection of positive clones on SD/‐Leu medium plates with different concentrations of aureobasidin A (AbA) at 30 °C for 3 d. The optimal concentration of AbA was determined to be 200 ng per mL, which was the lowest concentration that could completely inhibit strain growth for use in subsequent screening.

To confirm that FoxO interacts with the potential promoter regions of the *BOK*, *BNIP*, and *Bax* genes, the *FoxO* fragment was cloned into the pGADT7 vector to create the prey plasmid (pGADT7‐*FoxO*). The pGADT7‐*FoxO* plasmid was then transformed into the positively verified pAbAi‐pBOK, ‐pBNIP, and ‐pBax bait strains, followed by the selection of positive clones on SD/‐Ura medium plates with an AbA concentration of 200 ng per mL at 30 °C for 3 d.

### Prediction of BOK Promoter and EMSA

The promoter core region of the *BOK* gene was predicted using the JASPAR database (http://jaspar.genereg.net/).

For EMSA, the full‐length of *FoxO* was cloned into the pET‐28b(+) vector to construct plasmids expressing His‐FoxO. The recombinant proteins were expressed in *E. coli* strain BL21 and purified using NI‐NTA 6FF columns (Sangon Biotech, C600793‐0005). Cy5‐labeled oligonucleotide probes A (5′‐GGAGTTTTCTGTTTGTTTAGTAGTTTTATA‐3′), B (5′‐ATCATAAATATGTTTATTTATGATAATTTA‐3′), C (5′‐TTTTTAATTTATTAATTATTTTAATTTTAA‐3′), and D (5′‐TTATTTTTATGGTATTTACAAATTTTGTTA‐3′), corresponding to predicted FoxO binding regions A (‐1829/‐1819 bp), B (‐858/‐849 bp), C (‐629/‐619 bp), and D (‐132/‐121 bp) of *BOK*, were synthesized along with unlabeled competitor probes (Tsingke Biotech). To screen the potential FoxO binding regions of *BOK*, purified His‐FoxO proteins and Cy5‐labeled probes were mixed and incubated in EMSA/Gel‐Shift Binding Buffer (Beyotime, GS005) at 25 °C for 20 min. Then, the total mixtures were loaded onto a 6% polyacrylamide gel in Tris‐borate buffer and electrophoresed. After electrophoresis, the gel was analyzed using the LI‐COR Odyssey DLx Imaging System (LI‐COR, Lincoln, NE, USA).

To identify FoxO binding region B (‐858/‐849 bp) of *BOK*, purified His‐FoxO proteins, Cy5‐labeled probes, and an antibody against FoxO were mixed with a dilution series of unlabeled competitor probes in EMSA/Gel‐Shift Binding Buffer. The mixture of His‐tag and Cy5‐labeled probes in EMSA/Gel‐Shift Binding Buffer served as a negative control. The His‐tag in the EMSA/Gel‐Shift Binding Buffer served as a blank control. The total mixtures were analyzed by 6% polyacrylamide gel electrophoresis and visualized using the LI‐COR Odyssey DLx Imaging System.

### Dual‐Luciferase Reporter Assay

The full‐length of *FoxO* was cloned into the pGreenII 0029 62‐SK vector, and the fragment corresponding to −900–−700 bp of the *BOK* gene was cloned into the pGreenII 0800‐LUC vector. These constructs were individually transformed into *Agrobacterium tumefaciens* strain GV3101 and then introduced into the leaves of 5‐week‐old *Nicotiana benthamiana* plants. At 48 h post‐infiltration, samples of the plant tissues were examined using the Newton FT500 imaging system (Vilber, Collégien, France). The ratio of luciferase (LUC) to *Renilla* luciferase (REN) luminescence was measured using a Dual‐Luciferase Reporter Assay System (Promega, E1910). Three independent experiments were conducted, with three biological replicates in each experiment.

### ChIP In Vivo

Approximately 80 mg of pooled V^+^ female adults (≈50 individuals) were analyzed for the transcriptional activities of FoxO on the promoter of *BOK* using a SimpleChIP Plus Enzymatic Chromatin IP Kit (Magnetic Beads) (Cell Signaling Technology, Danvers, MA, USA; 9005S). In brief, leafhoppers ground in liquid nitrogen were fixed in 37% formaldehyde for 20 min, and crosslinking was quenched with 125 mm glycine for 5 min. The ground tissues were enzymatically digested and then sonicated extensively to shear the chromatin. The sheared chromatin was successively incubated with antibodies against FoxO or IgG and then with Protein G Magnetic Beads (Cell Signaling Technology, 9006). Purified immunoprecipitated BOK promoter from the IP samples was assayed by qPCR for quantitative analysis of the ChIP enrichment efficiency. Fold enrichment was calculated after normalizing to 1% input. Three biological replicates were assayed.

### Effect of Knocking Down BOK on Ovary Apoptosis, Offspring Fitness of V+ Females, and RGDV Maternal Transmission

The T7 RNA polymerase promoter sequence was added to the forward and reverse primers for the *BOK* gene at their 5′ termini to amplify a region of ≈368 bp. The T7 RiboMAX Express RNAi System was utilized to in vitro synthesize dsRNAs targeting *BOK* (dsBOK).

Newly emerged potential V^+^ females were microinjected with ≈60 ng dsBOK per insect. At 5 d post post‐injection, two mating combinations were established: (i) dsBOK‐treated V^+^ female × V^−^ male and (ii) dsGFP‐treated V^+^ female × V^−^ male. In each combination, virgin females and males mated one to one in glass tubes containing rice seedlings for 3 d. Then, the potential V^+^ females were collected for virus detection by RT‐PCR assay. At 6 d after the removal of females, 10 intact eggs produced by each pair of the two mating combinations were collected by dissecting rice seedlings for RGDV detection using RT‐PCR assay.

A pool of RNAs was isolated from 15 dsRNA‐treated V^+^ females or eggs produced by four pairs of each mating combination described above. Then, RT‐qPCR assays were conducted to assay the expression of *BOK*, *caspase‐2*, *caspase‐8*, *IAP*, and *P8* genes in these RNA samples. A pool of proteins from the ovaries of 10 V^+^ females or eggs produced by 12 pairs from each of the above mating combinations was analyzed for the expression of BOK, caspase‐8, and P8 proteins using western blot assays. A pool of DNAs from the ovaries of 20 V^+^ females or from eggs produced by five pairs was analyzed for chromosomal DNA fragmentation. Ovaries of 15 V^+^ females were analyzed for the accumulation of cytochrome C in mitochondria. At least three biological replicates were subjected to RT‐qPCR, western blot, chromosomal DNA fragmentation, and mitochondrial isolation assays.

At 6 d after the removal of females from mating combinations, eggs produced by each pair were harvested and counted to determine the total number. The development of 11 eggs was analyzed by light microscope, and the length and diameter of eggs were obtained using Capture 2.0 software. Then, the eggs of each pair were placed on pieces of water‐soaked filter paper and monitored to characterize their development and hatching. A total of 11 biological pairs were assayed.

To determine maternal transmission of RGDV, all eggs at the red‐eye stage that were produced by each pair over the course of 5 d were harvested to assay RGDV infection. A total of five mating pairs of each mating combination were analyzed, with three biological replicates.

### Recombinant Baculovirus Expressing Viral Proteins and GFP

The coding regions of RGDV *P2N*, *P2C*, *P8*, *Pns11*, and *GFP* were amplified with a reverse primer that contained the coding sequences of the His‐tag or GFP and a forward primer to create P2N‐, P2C‐, P8‐, Pns11‐, and GFP‐His constructs. These purified PCR products were cloned into the pfastBacI vector (Thermo Fisher Scientific) using an In‐Fusion HD Cloning Kit (Clontech, Mountain View, CA, USA) to construct the recombinant baculoviruses containing P2N‐, P2C‐, P8‐, Pns11‐, or GFP‐His. Recombinant bacmids were generated by transforming *E. coli* DH10Bac (Thermo Fisher Scientific) with the recombinant baculoviruses.

Sf9 cells (Thermo Fisher Scientific, 11496015) were transfected with purified recombinant bacmid in the presence of Cellfectin II (Thermo Fisher Scientific), according to the manufacturer's instructions. At 48 h post‐infection (hpi), the cells were harvested for cell viability tests using CCK‐8 kit (Beyotime, C0037). The absorbance of treated cells was measured at 450 nm with a Microplate spectrophotometer. Cell viability was calculated and normalized using the following formula: Cell viability (%) = 100 × (*A*450​(treatment)−*A*450​(blank)​)/(*A*450​(control) −*A*450​(blank)). At 48 hpi, cells were also harvested to examine the expression levels of P2N‐, P2C‐, P8‐, Pns11‐, and GFP‐His, as well as the accumulation of P85, AKT, p‐AKT, FoxO, BOK, and caspase‐8 proteins using western blot assays. Three biological replicates were evaluated.

To test the mitochondrial membrane potential of Sf9 cells expressing P2N‐His, a Mitochondrial Membrane Potential Assay Kit with Rhodamine 123 (Beyotime, C2008S) was used. In brief, ≈1 × 10^6^ Sf9 cells were harvested and suspended in PBS and then incubated with rhodamine 123 at a concentration of 1 µm in PBS at 37 °C for 1 h. The cells suspended in PBS were analyzed immediately using a flow cytometer. Data from three independent biological experiments were analyzed and visualized as a plot of fluorescence intensity of rhodamine (*x*‐axis) against cell number (*y*‐axis). Sf9 cells inoculated with an empty baculovirus vector served as negative controls, while cells treated with 10% H_2_O_2_ were used as positive controls.

### Effect of Prokaryotically Expressed P2N on Apoptosis in Ovaries and Eggs

The fragment of *P2N* and full‐length *GFP* were respectively cloned into the pET‐28b(+) vector to construct separate plasmids expressing His‐P2N and His‐GFP. Recombinant proteins were expressed in *E. coli* strain BL21 and purified using NI‐NTA 6FF (Sangon Biotech, C600793‐0005). Purified His‐P2N or His‐GFP proteins were microinjected into the newly emerged V^−^ females (≈2.5 mg per insect). At 5 d post‐injection, mating combinations were established between His‐P2N or His‐GFP‐treated virgin females and V^−^ males mated one to one in glass tubes containing rice seedlings for 3 d, after which the females and males were removed. At 6 d after the removal of females, intact eggs of each pair were harvested by dissecting rice seedlings.

A pool of RNAs from the ovaries of 15 females or from eggs produced by four pairs of mating combinations was analyzed for the expression of *ILP2*, *PTEN*, *PI3K*, *AKT*, *FoxO*, *BOK*, *caspase‐2*, *caspase‐8*, and *IAP* genes using RT‐qPCR assays. A pool of proteins from the ovaries of 10 females or from eggs of 12 mating pairs was analyzed for the expression of P85, AKT, p‐AKT, FoxO, BOK, and cleaved caspase‐8 proteins using western blot assays. A pool of DNAs from the ovaries of 10 females was assayed for chromosomal DNA fragmentation. At least three biological replicates were evaluated by RT‐qPCR, western blot, and chromosomal DNA fragmentation assays.

### Interaction of P2N with ILP2

For Y2H assays, a fragment of *P2N* was cloned into the pGADT7 vector as a prey plasmid. The full‐length open reading frame (ORF) of *ILP2* was cloned into pGBKT7 as a bait plasmid. The bait and prey were then co‐transformed into the AH109 yeast strain. The transformants were subsequently screened using SD/‐Leu‐Trp, SD/‐His‐Leu‐Trp, and SD/‐Trp‐Leu‐His‐Ade culture media. Positive clones were selected on SD/‐Trp‐Leu‐His‐Ade plates containing X‐α‐gal to evaluate their β‐galactosidase activity. The interaction between pGBKT7‐53 and pGADT7‐T was used as a positive control, whereas the interaction between pGBKT7‐Lam and pGADT7‐T was used as a negative control.

In the GST pull‐down assays, the full‐length ORF of *ILP2* was cloned into the pGEX‐4T‐3 vector to construct a plasmid expressing the GST fusion protein as bait (GST‐ILP2). The recombinant proteins fused with the GST tag and GST were separately expressed in *E. coli* strain BL21. Lysates were then incubated with glutathione‐sepharose beads (GE Healthcare, Uppsala, Sweden) and subsequently with recombinant proteins fused with a His tag. Eluted proteins were analyzed by western blot assay using GST‐ and His‐tagged antibodies.

### Co‐IP Assay

In the Co‐IP assays, purified His‐P2N was microinjected into ≈100 newly emerged V^−^ females (≈5 mg per insect). At 2 d post post‐injection, the treated females were ground with liquid nitrogen, and then lysed with RIPA Buffer (Thermo Fisher Scientific, 89901) for 10 min on ice. After centrifugation at 13400 × *g* for 10 min at 4 °C, the supernatant was collected. Next, 20 µL of BeyoMag Protein A+G beads (Beyotime, P2108), washed with PBS twice, were incubated with antibody against ILP2 or PTEN for 2 h at 4 °C. The incubation of beads with IgG (TransGen Biotech, HS101) served as a negative control. After being washed three times, the beads were incubated with the supernatant of His‐P2N‐treated females at 4 °C overnight. The beads were then washed in PBS and analyzed with western blot assays using an antibody against ILP2 or PTEN.

### ILP2 Expression in Tissues and its role in Offspring Fitness of V+ Females

Midguts or ovaries of 10 V^−^ newly emerged females, as well as hemolymph or fat bodies of 30 newly emerged adults, were dissected to assay their expression of ILP2 using RT‐qPCR or western blot assays. At least three biological replicates were evaluated.

To understand the effect of knocking down *ILP2* on offspring fitness of V^+^ females, at 6 d after the removal of females from the mating combination set up for dsILP2‐treated virgin V^+^ females × V^−^ males crosses, the total number of eggs produced per pair was counted. The development of 15 eggs was observed by light microscopy, and the length and diameter of eggs were obtained using Capture 2.0 software. Then, the eggs of each pair were placed on pieces of water‐soaked filter paper and monitored to observe their development and hatching. A total of 11 biological pairs were evaluated.

### Interaction of P2N with PTEN

For Y2H assays, a fragment of *CHK* or P2C was cloned into the pGADT7 vector to obtain prey plasmids. The full‐length ORF of *PTEN* and the fragment corresponding to the PTPc, C2, or C tail domain were cloned into pGBKT7 as a bait plasmid. Bait and prey plasmids were then co‐transformed into the AH109 yeast strain. Transformants were subsequently screened by culture on SD/‐Leu‐Trp, SD/‐His‐Leu‐Trp, and SD/‐Trp‐Leu‐His‐Ade culture media. Positive clones were selected on SD/‐Trp‐Leu‐His‐Ade plates containing X‐α‐gal to evaluate their β‐galactosidase activity. The interaction between pGBKT7‐53 and pGADT7‐T was used as a positive control, whereas the interaction between pGBKT7‐Lam and pGADT7‐T was used as a negative control.

In the GST pull‐down assays, the fragment of *CHK* was cloned into pET‐28b(+) to construct plasmids expressing the His‐tagged fusion protein as prey (His‐CHK). The full‐length ORF of *PTEN* or the fragment of C2 was cloned into the pGEX‐4T‐3 vector to construct plasmids expressing GST‐PTEN or GST‐C2. Recombinant proteins fused with the GST tag and GST were separately expressed in *E. coli* strain BL21. Lysates were then incubated with glutathione‐sepharose beads (GE Healthcare, Uppsala, Sweden) and subsequently with recombinant proteins fused with a His tag. Eluted proteins were analyzed by western blot assay using GST‐ and His‐tagged antibodies.

To generate the PTEN^Δ322^ or PTEN^Δ371^ fragment, the tyrosine residues at positions 322 or 371 of PTEN were substituted with histone. Then, these fragments, as well as *GFP*, *PTEN*, *P2N*, and *CHK* fragments, were amplified with a reverse primer that contained the Myc‐tag coding sequence to construct PTEN^Δ322^‐, PTEN^Δ371^‐, GFP‐, PTEN‐, P2N‐, and CHK‐Myc. These fragments were individually cloned into the pFastBacI vector to construct the recombinant baculoviruses. Recombinant bacmids were generated by transforming *E. coli* DH10Bac with the recombinant baculoviruses, which were then transfected into Sf9 cells.

For the protein phosphorylation analysis, total proteins were extracted from infected Sf9 cells with RIPA buffer (25 mm Tris, 150 mm NaCl, 1 mm EDTA, 1% NP‐40, 0.1% SDS, pH 7.4) including a protease inhibitor cocktail (Roche, Basel, Switzerland; 04693132001) and phosphatase inhibitor cocktail (MedChemExpress, Monmouth Junction, NJ, USA; HY‐K0022). The lysates were separated by SDS‐PAGE using gels including Phos‐tag acrylamide (Wako, Osaka, Japan; AAL‐107) and MnCl_2,_ and then transferred to PVDF membranes (0.2 µm; Millipore, Billerica, MA, USA; ISEQ00010) according to the Phos binding reagent (Phosbind) acrylamide protocol. The blocked membranes were incubated with Myc antibody for the PTEN phosphorylation assay.

### PIP2 or PIP3 Contents in Ovaries of V+ Females

To examine PIP2 or PIP3 protein contents of infected ovaries, a pool of ovaries of 15 V^+^ females of eggs from five mating pairs were analyzed by enzyme‐linked immunosorbent assay (ELISA) using the Bovine PIP2 ELISA Kit (Shanghai Xuanke Biotechnology, Shanghai, China; XK‐J2591) or Mouse PIP3 ELISA KIT (Shanghai Yuanju Biology, Shanghai, China; YJ174655) for each replicate. In brief, ground samples were incubated with antibodies immobilized in the reaction well provided by the kit for 1 h at 37 °C. After being washed three to five times, each reaction was incubated with Streptavidin‐HRP for 30 min at 37 °C. After being washed another three times, each reaction was incubated with substrates A and B for 10 min at 37 °C. The absorbance of samples at 450 nm was measured using the SPARK 10 m Microplate spectrophotometer. PIP2 or PIP3 content was calculated using a standard. Four replicates were evaluated.

### Effect of Knocking down PTEN on Offspring Fitness of V+ Females and Viral Maternal Transmission

To understand the effect of knocking down *PTEN* on offspring fitness of V^+^ females, at 6 d after the removal of females from the mating combination set up for dsPTEN‐treated virgin V^+^ female × V^−^ male crosses, the total number of eggs laid by each pair was counted. The development of 15 eggs was observed by light microscopy, and the length and diameter of eggs were obtained using Capture 2.0 software. Then, the eggs of each pair were placed on pieces of water‐soaked filter paper and monitored to observe their development and hatching. A total of 11 biological pairs were evaluated.

To determine maternal transmission of RGDV, all eggs at the red‐eye stage laid by each pair over the course of 5 d were harvested to assay RGDV infection. In total, five mating pairs of each mating combination were analyzed. Three biological pairs were assayed.

### Effect of RDV or RSV Infection on Apoptosis in Ovaries of *N. Cincticeps* or *L. Striatellus*

Second instar virus‐free nymphs of *N. cincticeps* and *L. striatellus* were allowed to feed for 3 d on RDV‐ or RSV‐infected rice plants, respectively, and then transferred to virus‐free rice seedlings. At 14 d padp, most insects had developed into adults. At 9 d after eclosion, the potential V^+^ females were microinjected with 120 nL of 10% H_2_O_2_. At 12 h post‐injection, the ovaries were dissected from the female bodies. These bodies were also individually assayed for virus infection by RT‐PCR assays.

To determine the effect of viral infection on apoptosis of ovary tissues, the ovaries from four and 30 confirmed virus‐infected females were analyzed for caspase‐3 activity and chromosomal DNA fragmentation, respectively, at 9 d after eclosion. At least three replicates were assayed.

Total proteins from the ovaries of 30 virus‐infected females were examined using western blot assays for caspase‐8 and capsid protein P8 of RDV or CP of RSV. Three biological replicates were assayed.

### Phylogenetic Analyses

MEGA 11.0 program was conducted to analyze phylogenetic relationships based on the comparison of the amino acid sequence of PTEN with homologues in other insect species. The available sequences were aligned using ClustalW. Phylogenetic trees were reconstructed using the neighbor‐joining method with P‐distance, and gaps or missing data were treated with complete deletion. The reliability of the neighbor‐joining trees was estimated by bootstrap analysis with 1000 replicates.

### Structural Modeling and Molecular Docking

The protein structures of P2, ILP2, and PTEN were predicted using AlphaFold3 (DeepMind, UK). Molecular docking simulations were performed using GRAMM‐X (http://vakser.compbio.ku.edu/resources/gramm/grammx/) with default parameters. The top‐scoring protein complexes were subsequently analyzed for protein‐protein interactions using PDBePISA (https://www.ebi.ac.uk/pdbe/pisa/). 3D structures were visualized and analyzed using PyMOL v3.1 (Schrödinger, LLC).

### Statistical Analysis

Two‐tailed Student's *t*‐tests (for comparisons between two groups) were conducted using GraphPad Prism 9 software (GraphPad Software, Boston, MA, USA) to quantitatively analyze RT‐qPCR assays, caspase‐3 activity, egg development, maternal transmission, ChIP‐qPCR, relative LUC/REN, cell viability, rhodamine 123 intensity, and the PIP2/PIP3 ratio. Data were also graphed using GraphPad Prism 9.

Multi‐variate analysis was conducted using SPSS version 21 (IBM Corp., Armonk, NY, USA) to assess the difference in viability of Sf9 cells, relative transcript level of ILP2 among tissues, and the caspase‐3 activity level in ovaries of *N. cincticeps* and *L. striatellus*. Multiple comparisons of means were performed using Tukey's honestly significant difference test following one‐way analysis of variance. Any comparisons with *P*‐values less than 0.05 were considered statistically significant. Data were presented as Means (±SD).

## Conflict of Interest

The authors declare no conflict of interest.

## Author Contributions

H.W. and W.W. contributed equally to this work. The conceptualization is carried out by T.W. and Q.C. The methodology is developed by H.W. and W.W. The investigation is performed by H.W., W.W., Q.L., H.Y., and C.L. The original draft is written by Q.C., while the review and editing are completed by T.W. and Q.C. Funding acquisition is handled by Q.C., and resources are provided by T.W. Supervision is conducted by T.W. and Q.C.

## Supporting information



Supporting Information

## Data Availability

All data are available in the main text or the supplementary materials.
