# Supplementary material for: Plant Virus‐Induced Inheritable Apoptosis Drives Reproductive Costs in Female Insect Vectors to Balance Viral Biparental Transmission
Source: Adv Sci (Weinh). 2025 Nov 12;13(5):e05681. doi: 10.1002/advs.202505681 (PMC12849885; doi:10.1002/advs.202505681)
Supplement: Supplementary file 1 — Supporting Information [file ADVS-13-e05681-s001.pdf]

Supporting Information

Plant Virus-Induced Inheritable Apoptosis Drives Reproductive Costs in Female Insect Vectors to Balance Viral Biparental Transmission

*Haibo Wu#, Wenqiang Wan#, Qingquan Liang, Hengsong Yang, Chengcong Lu, Taiyun Wei, Qian Chen**

State Key Laboratory of Agricultural and Forestry Biosecurity, Fujian Agriculture and Forestry University, Fuzhou, Fujian, China

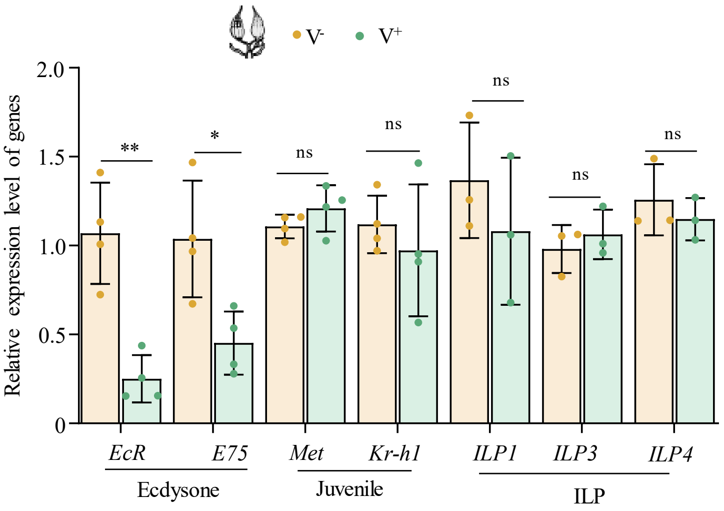

Figure S1 RGDV infection of females increased expression of genes related to ecdysone, juvenile hormone, and ILPs in ovaries. Statistical analyses were performed using Two-tailed Student's *t*-tests. Error bars show the SD; $n_{ovary} = 30$, with four biological replicates. *, $p < 0.05$; **, $p < 0.01$; ns, not significant.

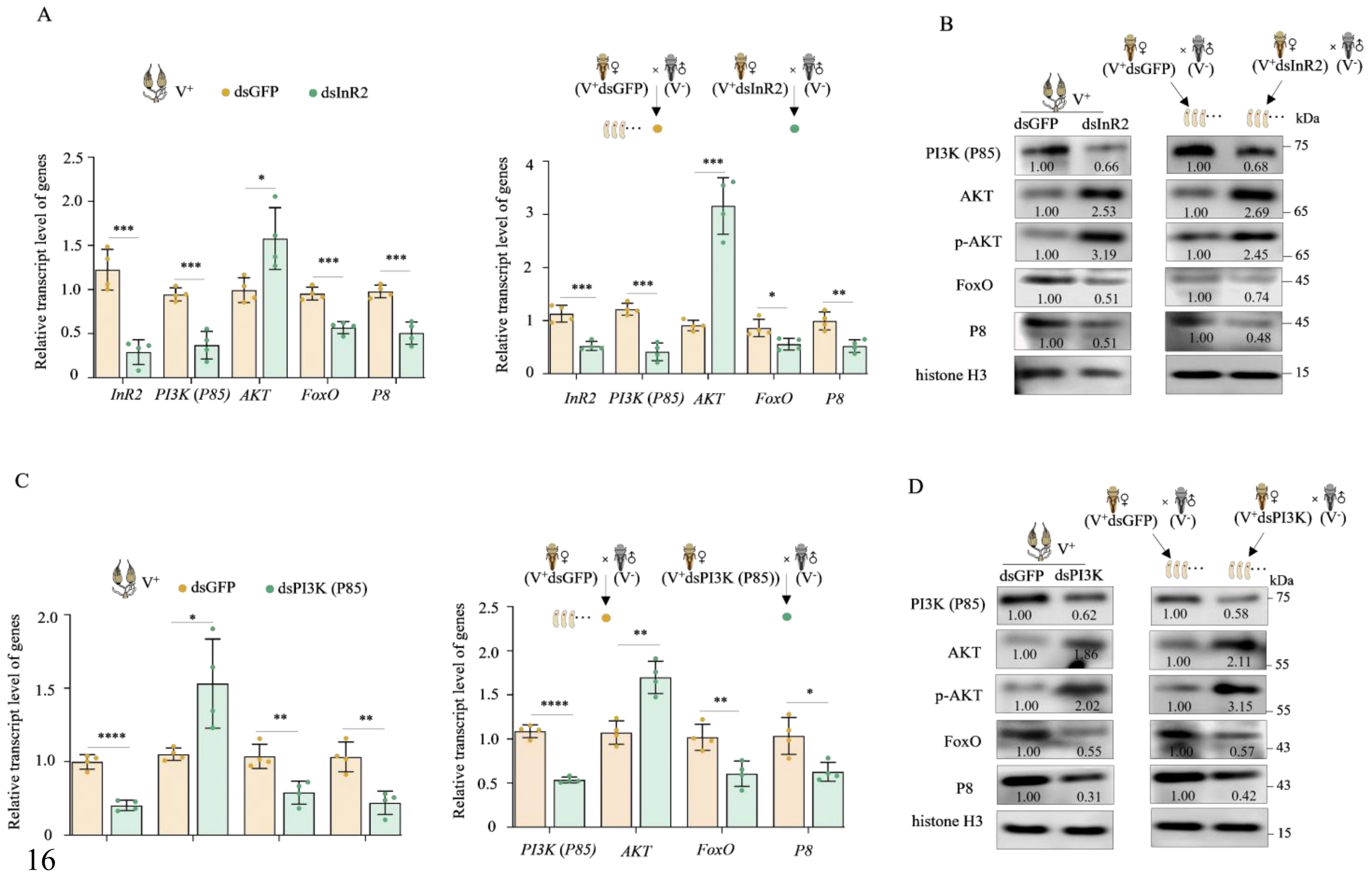

Figure S2 Knocking down *InR2* or *P85* of PI3K in V+ females promoted FoxO signaling and RGDV infection in ovaries and eggs. (Error bars show the SD; $n_{ovary} = 30$, n_{egg} = all eggs produced by four pairs of each mating combination, four biological replicates in A and C; $n_{ovary} = 20$, n_{egg} = all eggs produced by 12 pairs of each mating combination, three biological replicates in B and D). Statistical analyses of A and C were performed using Two-tailed Student's *t*-tests. Error bars show the SD. *, $p < 0.05$; **, $p < 0.01$; ***, $p < 0.001$; ****, $p < 0.0001$.

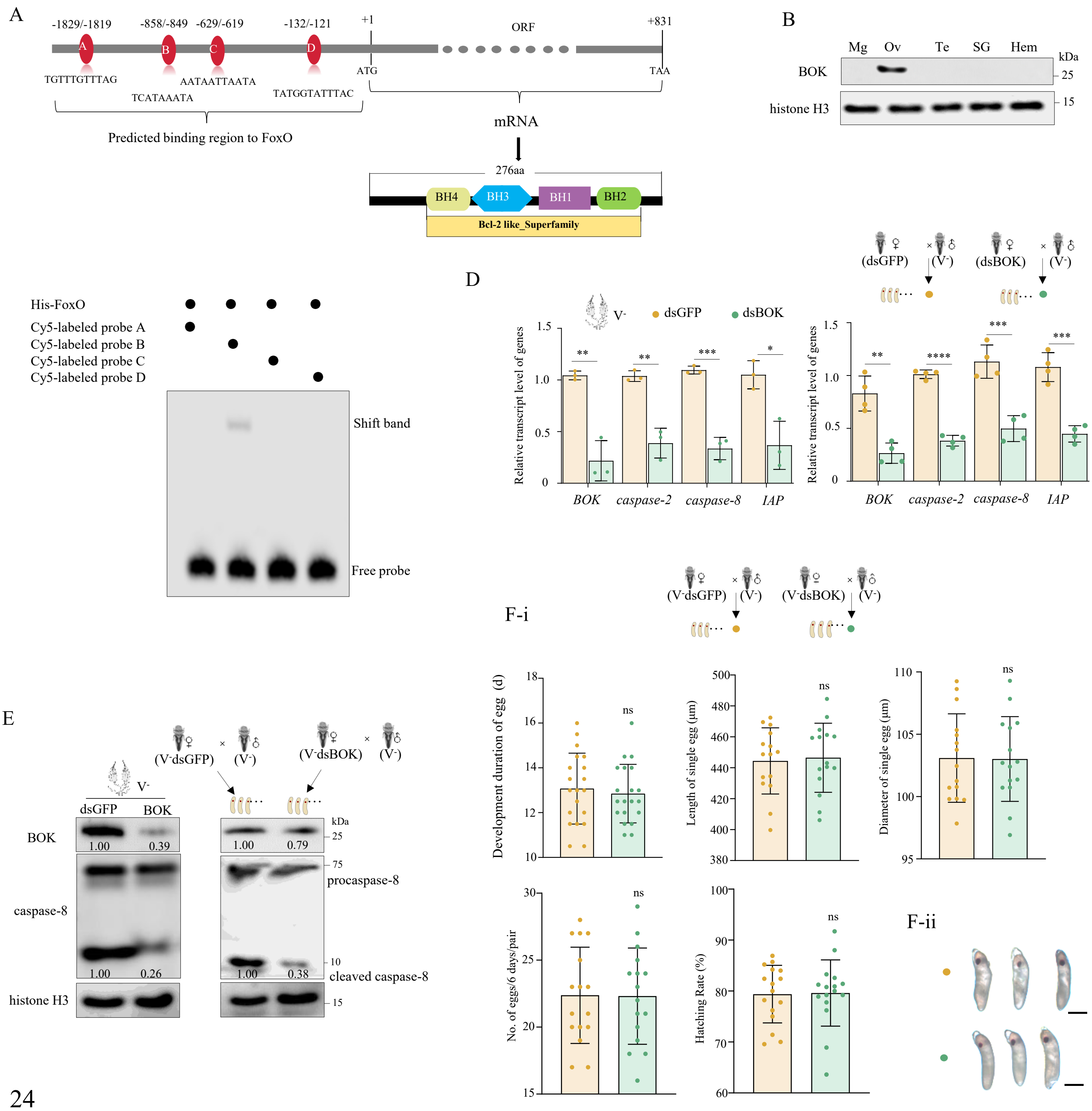

Figure S3 BOK regulating the apoptosis of ovaries. A) Schematic illustration describing genome, mRNA, and protein conserved domains of BOK, as well as its predicted FoxO binding region. **B)** BOK accumulation in ovaries of leafhoppers (n *leafhopper* = 10, three biological replicates). **C)** Preliminary EMSAs showing the binding of FoxO to region B of *BOK*. **D** and **E)** Knocking down *BOK* in V- females reduced the gene expression and protein accumulation of BOK, caspase-2, caspase-8, and IAP in ovaries and eggs produced by dsRNA-treated V- female × V- male crosses (n ovary = 30, three biological replicates in d; n ovary = 20, n egg = all eggs produced by 12 pairs of each mating combination, three biological replicates). **F)**

Knocking down *BOK* in V$^-$ females had a limited effect on progeny egg number, hatching rate, size and development (n_{pair}= 1, at least 15 biological replicates). Bars, 300 µm. Statistical analyses of D and F-i were performed using Two-tailed Student's *t*-tests. Error bars show the SD. *, $p < 0.05$; **, $p < 0.01$; ***, $p < 0.001$; ns, not significant.

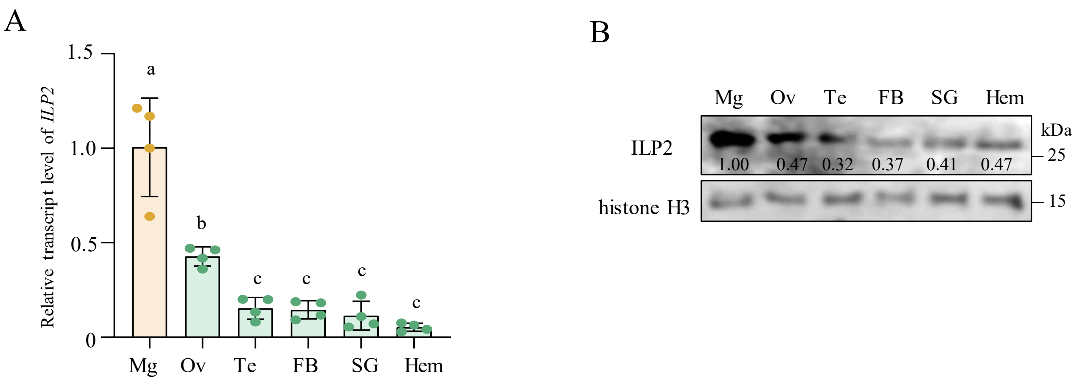

Figure S4 Expression of ILP2 in tissues of leafhoppers, as determined by RT-qPCR (A) and western blot (B) assays. Error bars show the SD; $n_{\text{midgut}} = 10$, $n_{\text{ovary}} = 20$; $n_{\text{hemolymph}} = 30$, $n_{\text{fat body}} = 30$, four biological replicates in A and three biological replicates in B. The significance of differences was determined using Tukey's HSD test at a $p < 0.05$ threshold. Different letters above columns indicate that the means differ significantly. Mg, midgut. Ov, ovary. Te, testis. FB, fat body. SG, salivary gland. Hem, hemolymph.

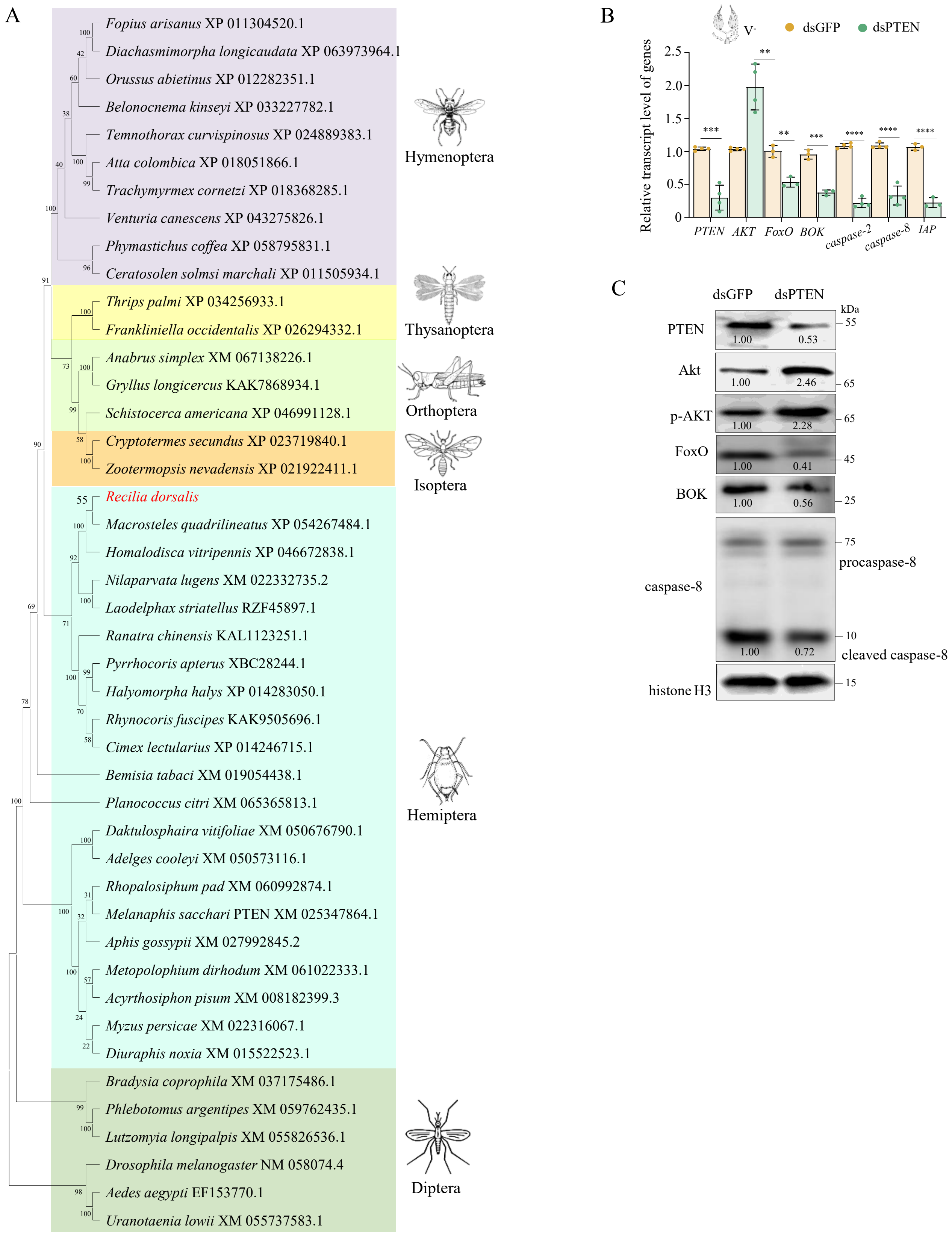

Figure S5 PTEN positively regulated the apoptosis of ovaries. A) Phylogenetic relationship of PTEN with homologues in other insect species using MEGA 11. **B)** and **C)** Knocking down *PTEN* in V^{-} females inhibited the FoxO signaling axis and apoptosis in ovaries and eggs.

Statistical analyses were performed using Two-tailed Student's *t*-tests. Error bars show the SD; $n_{\text{ovary}} = 30$, n_{egg} = all eggs produced by four pairs of each mating combination, four biological replicates in B; $n_{\text{ovary}} = 20$, n_{egg} = all eggs produced by 12 pairs of each mating combination, three biological replicates in C; **, $p < 0.01$; ***, $p < 0.001$; ****, $p < 0.0001$.

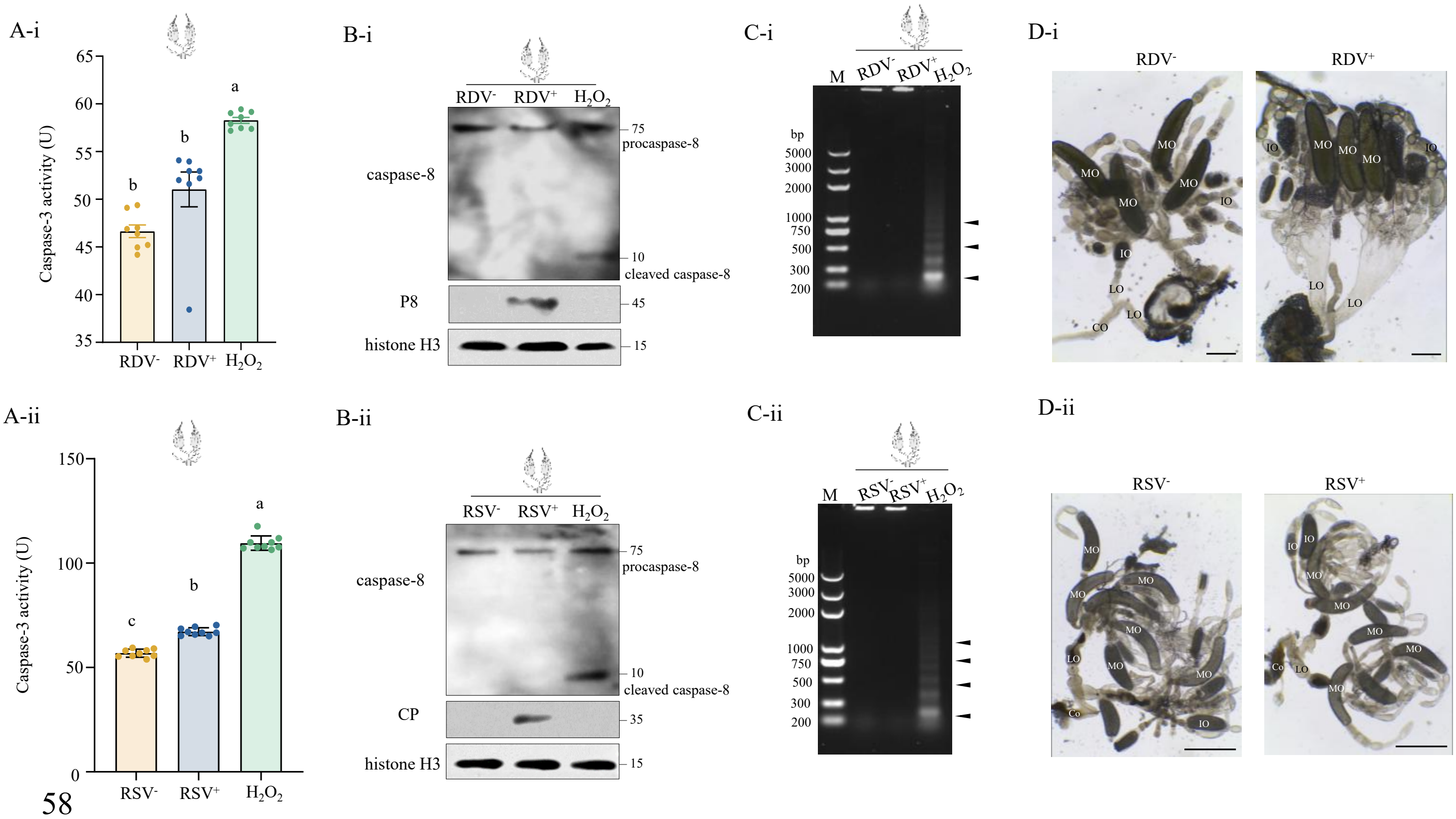

Figure S6 Other rice viruses that are exclusively maternally transmitted failed to induce apoptosis in ovaries of their insect vectors. A) RDV or RSV infection had a limited effect on caspase-3 activity of ovaries from *N. cincticeps* or *L. striatellus* females, respectively (n_{ovary} = 4, eight biological replicates). The significance of differences was determined using Tukey's HSD test at a $p < 0.05$ threshold. Different letters above columns indicate that the means differ significantly. **B)** RDV or RSV infection had limited effect on activation of caspase-8 in ovaries derived from *N. cincticeps* or *L. striatellus* females (n_{ovary} = 30, three biological replicates). Bands of histone H3 indicate the loading of equal amounts of protein. P8, outer capsid protein of RDV. CP, coat protein of RSV. **C)** RDV or RSV infection had limited effect on chromosomal DNA fragmentation of ovaries derived from *N. cincticeps* or *L. striatellus* females (n_{ovary} = 30, three biological replicates). Lane M, DNA marker. **D)** Effect of RDV or RSV infection on ovary development. RDV+, RDV-infected; RDV-, uninfected; RSV+, RSV-infected; RSV-, uninfected; MO, mature oocyte; IO, immature oocyte; LO, lateral oviduct; CO, common oviduct. Bars, 300 µm.
